# Evolutionary evidence on suitability of SecD as a target for development of antibacterial agents against *Staphylococcus aureus*

**DOI:** 10.1002/ece3.1951

**Published:** 2016-02-03

**Authors:** Shaomin Yan, Guang Wu

**Affiliations:** ^1^ Guangxi Bioscience and Biotechnology Research CenterGuangxi Academy of Sciences98 Daling RoadNanningGuangxi530007China

**Keywords:** Antibacterial agent, bacteria, evolution, multidrug resistance, SecD, secretion system, *Staphylococcus aureus*

## Abstract

*Staphylococcus aureus* causes many infections and its drug resistance is a worrying challenge for medical care. The SecD subunit of Sec secretion system in methicillin‐resistant *S. aureus* is an attractive target because SecD dysfunction leads to the death of bacteria and SecD as a target is more efficient than SecA and SecF. Evolution could have made SecD to become insensitive to antibacterial agents although the drugs directly against SecD have yet to develop. So far, no detailed information on SecD evolution has been available, thus 2686 SecD sequences with full taxonomic information from kingdom to species were analyzed. First, the variance of pairwise *p*‐distance was evaluated for each taxonomic group. Second, the variance was further partitioned into intergroup and intragroup variances for quantification of horizontal and vertical gene transfer. Third, phylogenetic tree was built to trace the evolutionary pathway. The results showed that overall evolution of SecDs appears to have undergone horizontal and vertical gene transfer. Only 0.5% horizontal transfers were found between any two SecDs in *S. aureus*, 6.8% and 8.8% horizontal transfers were found between any two *Staphylococcus* SecDs from different and the same species, and only one SecD from *S. aureus* was located far away from its sister cluster. Thus, statistic and evolutionary analyses demonstrate that the SecDs from *staphylococcus* species have a small chance of mutating, and provide taxonomic evidence to use the SecD as a potential target for new generation of antibacterial agents against *S. aureus*.

## Introduction

*Staphylococcus aureus* is a Gram‐positive bacterium causing many infections. A worrying issue is methicillin‐resistant staphylococcal infections, which account for a half staphylococcal infections (Bassetti and Righi 2013), but the methicillin‐resistant *S. aureus* with vancomycin resistance is more challenging (Tarai et al. 2013). Apart from multidrug resistance, *S. aureus* secretes various virulence factors, such as exfoliative toxin D, toxic shock syndrome toxin, etc. (Schlievert et al. 2010), which play the roles of adhesion, invasion, and cytotoxicity to host cells (Quiblier et al. 2013). For virulent bacteria, secretion is more significant for humans not only because secreted pathogens lead to various diseases (Fagerlund et al. 2010), but also because multidrug resistance is closely related to secretion systems (Quiblier et al. 2011). Currently, Gram‐positive bacteria have six secretion systems: Sec secretion system, twin arginine targeting (Tat) secretion system, fimbrillin‐protein exporter (FPE), flagellar export apparatus (FEA), holins, and WXG100 secretion system (Wss) (Yuan et al. 2010). Most virulence factors are secreted through Sec secretion system (Driessen and Nouwen 2008).

A secretion system is composed of a number of proteins, and the knowledge on Sec secretion system mainly comes from Gram‐negative bacteria (Papanikou et al. 2007), which have at least seven secretion systems and Sec secretion system belongs to type II secretion system. There are seven subunit proteins, SecA, SecB, SecD, SecE, SecF, SecG, and SecY, in bacterial Sec system (Lycklama et al. 2012; Chatzi et al. 2013). SecE, SecG, and SecY gather together as a SecYEG complex forming a channel across the inner membrane, while SecG is not absolutely necessary but increases the translocation efficiency (Hanada et al. 1994). SecD and SecF construct a SecDF complex preventing preproteins from sliding backward from the translocation channel (Duong and Wickner 1997). SecA is an ATP‐dependent motor driving the stepwise translocation of secreted protein that forms a large complex with SecYEG and SecDF (Bauer et al. 2014) while SecB brings the precursor of secreted protein to SecA (Bechtluft et al. 2010).

On the other hand, Sec secretion system belongs to the resistance‐nodulation‐cell division (RND) family of multidrug exporters (Quiblier et al. 2011). Because Sec secretion system plays a central role in secretion of virulence factors and in multidrug resistance, it was proposed that the subunits of Sec secretion system could serve as targets for new generation of antibacterial drugs (Segers and Anne 2011; Rao et al. 2014). This is a new mechanism because the accumulated unsecreted proteins would lead to the death of bacteria (Sabate et al. 2010).

SecA captures considerable attention as a potential target for the development of new antibacterial drugs because it pushes secreted proteins across membrane (Bauer et al. 2014). SecB is less favorable because its position is not fixed on membrane (Bechtluft et al. 2010). Induction of SecY antisense RNA prevents the growth of cells that retain the integrated plasmid and selects for the cells that have lost the plasmid (Bae and Schneewind 2006). Meanwhile, SecDF is also an attractive target for the development of new antibacterial drugs (Quiblier et al. 2011, 2013) because it works at final stage of the secretion and pulls secreted proteins across the membrane (Lycklama et al. 2012). In particular, SecDF slows down the back and forward movement of secreted proteins inside SecYEG channel (Nouwen et al. 2005). Consequently, the deletion of either SecD or SecF resulted in a severe defect in secretion in vivo (Pogliano and Beckwith 1994b), and a decrease of membrane‐inserted SecA (Eichler and Wickner 1997). By contrast, overexpression of SecDF led an opposite effect (Kim et al. 1994; Pogliano and Beckwith 1994a). Along this line of evidence, designing of antibacterial agents against SecDF is indirectly to target SecA.

As a result, SecDF was proposed to serve as a potential target for the development of antibacterial drugs against *S. aureus* (Quiblier et al. 2011, 2013). Especially, both SecD and SecF are absent from eukaryotes but are not present in all prokaryotes (Economou 2001; Cao and Saier 2003), hence their specificity could be guaranteed. Indeed, not only SecDF acts together and has 12 transmembrane segments and two large periplasmic domains (Tsukazaki et al. 2011), but also their codes are located in a single open reading frame in many species (Bolhuis et al. 1998). In fact, there are only 10–30 copies of SecDF per cell, which are 10 times less abundant than SecYEG (Pogliano and Beckwith 1994b), so targeting of SecDF would not require a huge amount of antibacterial agents.

Take a step further, it is necessary to decide which is the best candidate among SecDF, SecD, and SecF, for the reason that SecDF exists in separate forms (Zhou et al. 2014). The accumulated evidence weighs more on SecD rather than SecF. For example, cells are unable to maintain a proton electrochemical gradient across the membrane in the absence of SecD and SecE (Nouwen et al. 1996). Besides, YajC, a small single membrane‐spanning protein, is also encoded in secD operon, and is associated with SecF and SecYEG (Nouwen and Driessen 2002) although YajC is not essential for protein translocation (Pogliano and Beckwith 1994a). Nevertheless, SecF can stabilize SecY and SecD (Sagara et al. 1994), and SecD is dysfunctional by binding of antibody to its periplasmic regions (Matsuyama et al. 1993). Ideally, antibacterial agents could aim at SecDF or SecD or SecF because different efficacies may be reached. In any case, no matter whether SecDF or SecD or SecF would be chosen as a target, the specificity of antibacterial agents is subject to mutations because any mutation would result in a changed structure in target (Nouwen et al. 2005) leading to reduced function of antibacterial agents (Foweraker et al. 2009).

**Figure 1:**
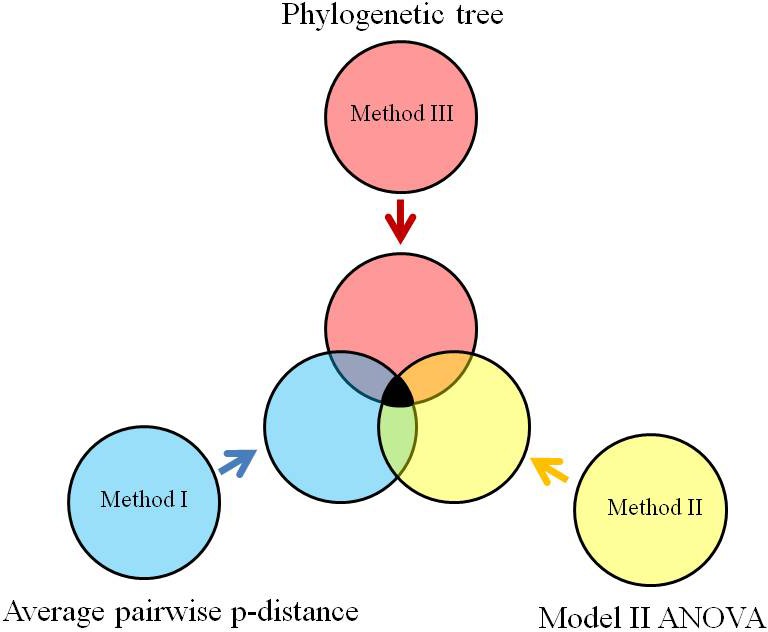
Determination of specificity of SecD as a potential target for development of antibacterial agents.

Of various approaches in vivo, in vitro and in silico, the analysis of variances and phylogenetics along taxonomic lineage is certainly helpful to understand the specificity of antibacterial agents. This is because a large taxonomic group would have more variants than a small taxonomic group does (Darwin 1872), so a target in a taxonomic group with less variants would be more specific. Yet, a small horizontal gene transfer implies a small variance between taxonomic groups (Sokal and Rohlf 1995), so a target in a taxonomic group with small variance between taxonomic groups would be more specific. Moreover, a taxonomic group spreads widely in phylogenetic tree would suggest an evolution in multiple times (Schombur et al. 2013), so a target in a taxonomic group that does not spread widely in phylogenetic tree would be more specific. As a whole, these three methods would approach to the specificity of antibacterial agents differently and provide different preferences, whose intersection suggests the possibility to let SecD as a target for developing new antibacterial agents to combat *S. aureus* as demonstrated in Figure 1.

## Materials and Methods

### Data

A total of 10,214 SecD protein sequences were downloaded from UniProtKB (The UniProt Consortium 2010), and this amount was all available SecD sequences in UniProtKB until June 11, 2014. Of the 10,214 SecD sequences, 10,003 SecD sequences were not marked as fragment, whose average length is 633 ± 160 (mean ± SD), and 211 SecD sequences were marked as fragment, whose average length is 288 ± 220, so the SecD sequences marked as fragment were excluded from this study.

In order to accurately, precisely, and reliably track the SecD evolutionary pathways, only the SecD sequences that have full taxonomic classification from superkingdom to kingdom, to phylum, to class, to order, to family, to genus and finally to species were included in statistical and phylogenetic analyses in this study. For this reason, the taxonomic lineage of the 10,003 SecD sequences was verified against various databases. This verification revealed that 170 SecD sequences from archaea and 5920 SecD sequences from bacteria had been fully documented with taxonomic information. After deletion of identical SecD sequences, the dataset with full taxonomic lineage from kingdom to phylum, to class, to order, to family, to genus, and finally to organism reduced to 162 SecD sequences from archaea and 2524 SecD sequences from bacteria (Table S1). The 162 SecD sequences from archaea covered one phylum, six classes, eight orders, 14 families, 56 genera, and 156 organisms (Table S2). The 2524 SecD sequences from bacteria covered 14 phyla, 29 classes, 66 orders, 142 families, 463 genera, and 2294 organisms (Table S3).

### Pairwise *p*‐distance in each taxonomic group

The 2686 SecD sequences can generate very precisely and accurately statistical estimates in each taxonomic group. Accordingly, the average pairwise *p*‐distance was computed using Mega software (Tamura et al. 2013) for each kingdom, phylum, class, order, family, and genus.

### Partitioning of inter‐ and intrataxonomic group variances

Model II ANOVA in SigmaStat (Systat Software 2003) was used to partition the variance of *p*‐distance in terms of inter‐ (between) phyla, classes, orders, families, genera, and intra‐ (within) phylum, class, order, family, genus, because of its particular suitability (Sokal and Rohlf 1995; Wu et al. 1999; Yan and Wu 2009, 2010a,b, 2011, 2013a,b).

### Phylogenetic tree

The alignment of SecD sequences with respect to bacteria and archaea was conducted using ClustalX (Larkin et al. 2007). The phylogenetic tree was constructed using ClustalX with neighbor‐joining method maximum likelihood, and then validated by using 1000 bootstrap replicates in ClustalX. The phylogenetic trees were presented using NJPlot (Perrière and Gouy 1996).

## Results

**Figure 2:**
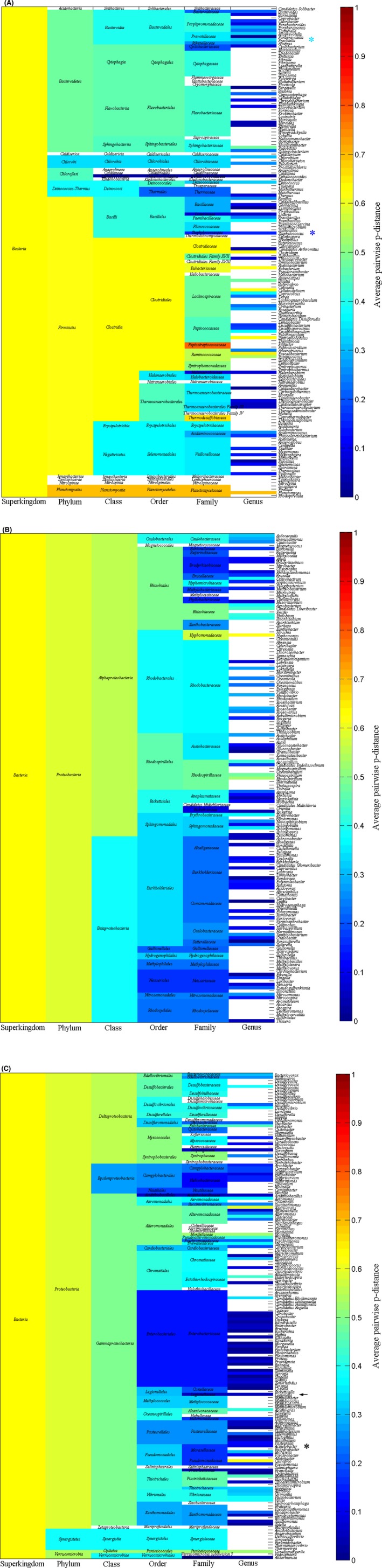
(A–C) Average pairwise *p*‐distance of SecDs in each taxonomic group of bacteria.

**Figure 3:**
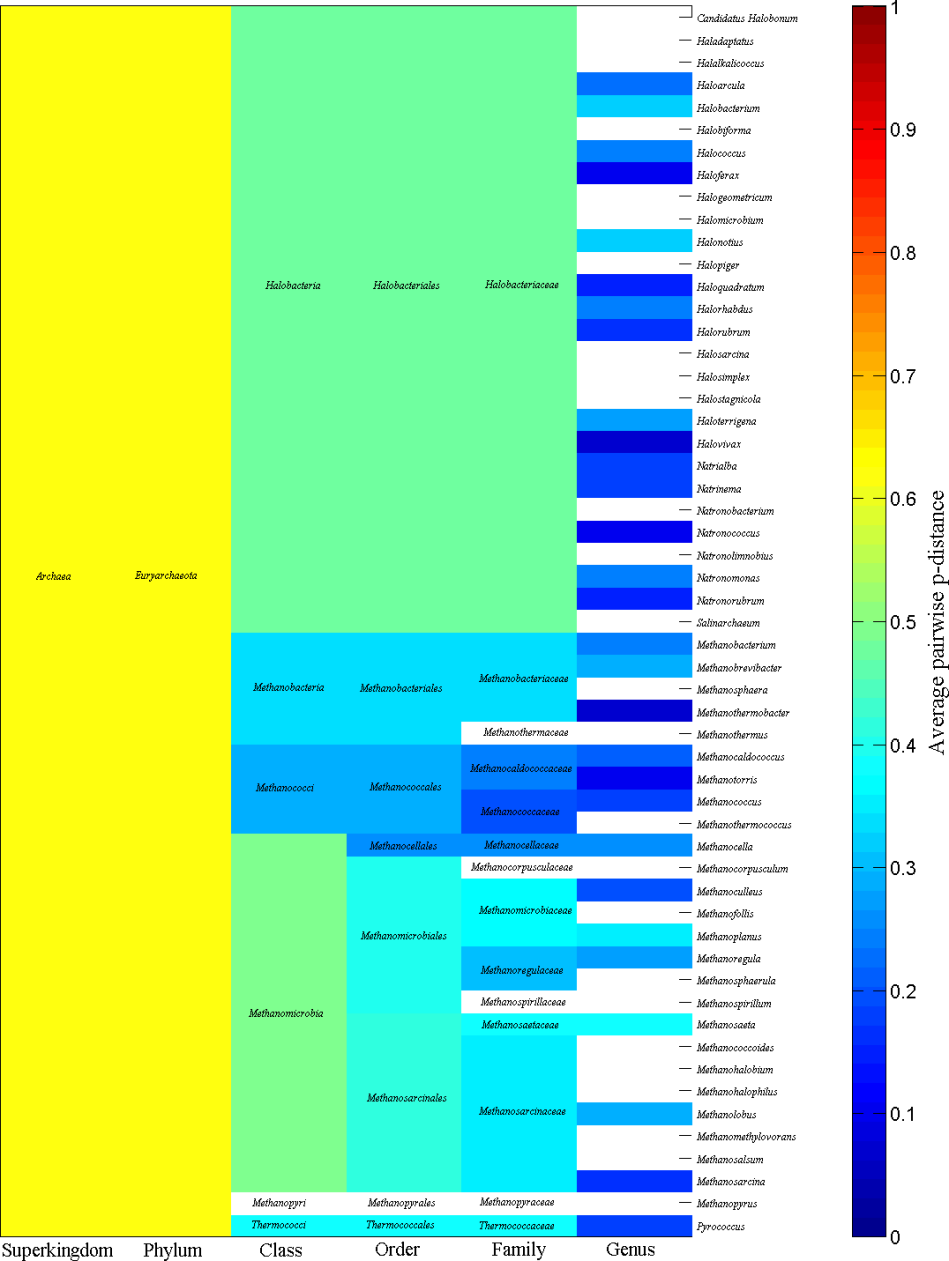
Average pairwise *p*‐distance of SecDs in each taxonomic group of archaea.

Figure 2 shows average pairwise *p*‐distance in each taxonomic group of bacteria, which cover 14 phyla, 29 classes, 66 orders, 142 families, and 463 genera. Figure 3 displays the average pairwise *p*‐distance in each taxonomic group of archaea. Although multidrug resistance is not involved in archaea, Figure 3 is useful for comparison. Staphylococcal species belongs to genus *Staphylococcus*, whose average pairwise *p*‐distance is 0.1562 (the deep blue colored cell in column genus indicated by dark blue asterisk in Fig. 2A), i.e., 15.62% differences would be expected when comparing any two SecD sequences from genus *Staphylococcus*. Comparing with other genera, the average pairwise *p*‐distance for *Staphylococcus* is really not large as seen in deep blue color. Still, *Prevotella* has an average pairwise *p*‐distance of 0.3281 (the eleventh cell from top in genus column indicated by light blue asterisk in Fig. 2A). This is interesting because methicillin‐resistant *S. aureus* could come from veterinary sources (Hiramatsu et al. 2013), so a large average pairwise *p*‐distance in *Prevotella* raised the question of whether the SecD in *Prevotella* is subject to horizontal gene transfer as methicillin‐resistant gene did. Furthermore, *Legionella* has an average pairwise *p*‐distance of 0.0521 (the deep blue colored cell in column genus indicated by black asterisk in Fig. 2C), and *Acinetobacter* has an average pairwise *p*‐distance of 0.0949 (the deep blue colored cell in column genus indicated by black arrow in Fig. 2C), which are far smaller than the abovementioned bacteria. These four genera account for 7.33% of total genera and significantly statistical difference exist between any two of these four genera (*P *<* *0.05). At species level, average *p*‐distances for *S. aureus*,* Legionella pneumophila,* and *Acinetobacter baumannii* are 0.0051, 0.0018, and 0.0261 respectively.

**Figure 4:**
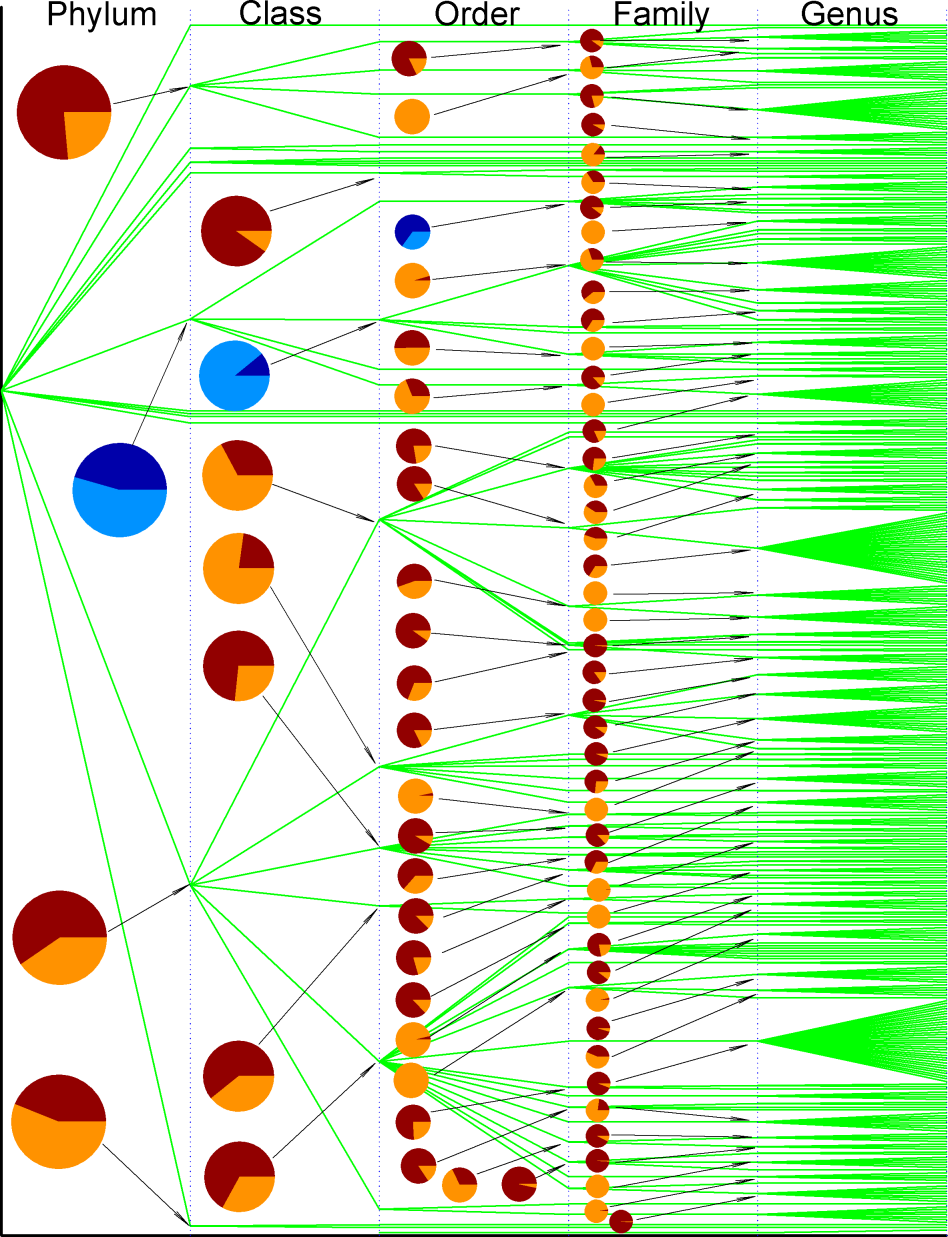
Partitioning of pairwise *p*‐distance into inter‐ and intragroup variances along taxonomic lineage of bacterial SecDs. The bifurcation is the point across taxonomic boundary line. Pies show intergroup variance (dark color) and intragroup variance (bright color). Taxonomic names can be found in Table S3. The pies marked in blue color are examples explained in text.

**Figure 5:**
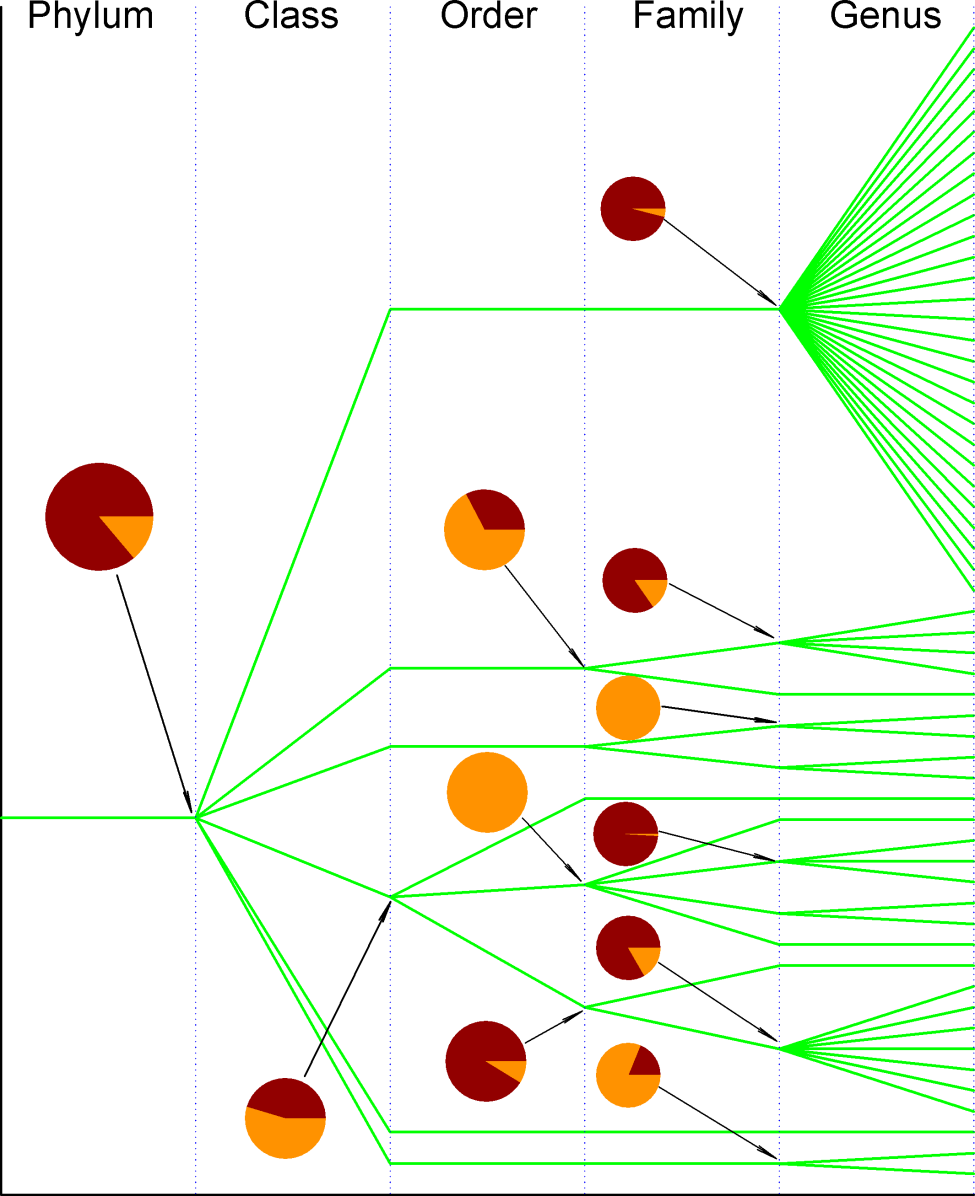
Partitioning of pairwise *p*‐distance into inter‐ and intra‐group variances along taxonomic lineage of archaeal SecDs. The bifurcation is the point across taxonomic boundary line. Pies show intergroup variance (dark color) and intragroup variance (bright color). Taxonomic names can be found in Table S2.

Figures 4 and 5 illustrate the partitioning of variance of pairwise *p*‐distance into inter‐ and intragroup variances in each taxonomic group of bacteria and archaea. These figures can be read as follows: the larger is the bright color area, the larger is the intragroup variance, the smaller is the intergroup variance, and the sum of inter‐ and intragroup variances is 100%. *S. aureus* belongs to genus *Staphylococcus*, family *Staphylococcaceae*, order *Bacillales*, class *Bacilli*, and phylum *Firmicutes*. The inter‐ and intraclass variances in phylum *Firmicutes* are outlined in the second pie marked in blue color from the top in column phylum in Figure 4. Phylum *Firmicutes* includes four classes, *Bacilli*,* Clostridia*,* Erysipelotrichia,* and *Negativicutes*, so this pie is associated with four lines. The pairwise *p*‐distance for these four classes was partitioned using model II ANOVA, which results in 45.59% interclass variance and 54.41% intraclass variance, and intra‐ and interclass variances can be viewed as vertical and horizontal gene transfers in SecD. However, class *Clostridia* embraces four orders *Clostridiales*,* Halanaerobiales*,* Natranaerobiales,* and *Thermoanaerobacterales*, and its inter‐ and intraorder variances are 10.90% and 89.11% (the second pie marked in blue color from the top in column class in Fig. 4), whereas class *Bacilli*, to which *S. aureus* belongs, contains just a single order *Bacillales*, thus no inter‐ and intraorder variances can be found for class *Bacilli*. In light of horizontal gene transfer, order *Bacillales* has such tendency because its interfamily variance is larger than its intrafamily variance (65.11% vs. 34.89%, the third pie marked in blue color from the top in column order in Fig. 4), i.e., the chance of horizontal gene transfer is larger than the chance of vertical gene transfer for the following six families, *Bacillaceae*,* Listeriaceae*,* Paenibacillaceae*,* Planococcaceae*,* Staphylococcaceae,* and *Thermoactinomycetaceae*. At family level, *Bacillaceae* is in favor of vertical gene transfer because its intergenus variance is smaller than its intragenus one (34.35% vs. 65.65%, the 6th pie in column genus in Fig. 4). By clear contrast, family *Paenibacillaceae* has a larger chance of horizontal gene transfer because its inter‐genus variance is remarkably larger than its intragenus one (89.77% vs. 10.23%, the 7th pie in column genus in Fig. 4). In this manner, the chances of horizontal and vertical gene transfers in the past are uncovered using model II ANOVA. Family *Staphylococcaceae* in currently available data has only a single genus *Staphylococcus*, which requires no horizontal gene transfer between genera. Accordingly, horizontal gene transfer for SecD in *S. aureus* can only occur either between families or between species or both. Because methicillin‐resistant *S. aureus* acquired mecA gene by intraspecies gene transfer (Baba et al. 2002), it could be possible that transmission of SecD could occur between species. Table 1 lists the partitioning of species variances in four genera that have a close relationship with multidrug resistance. As can be seen, genus *staphylococcus* has the smallest interspecies variance, which implies a possible intraspecies gene transfer as mecA did (Baba et al. 2002).

**Table 1:** Partitioning of species variances in four genera

Genus	Number of SecDs	Number of species	Average *p*‐Distance	Interspecies variance (%)	Intraspecies variance (%)
*Acinetobacter*	79	29	0.0949	61.78	38.22
*Legionella*	6	3	0.0521	99.70	0.30
*Prevotella*	38	22	0.3281	58.21	41.79
*Staphylococcus*	44	11	0.1562	56.29	43.71

**Figure 6:**
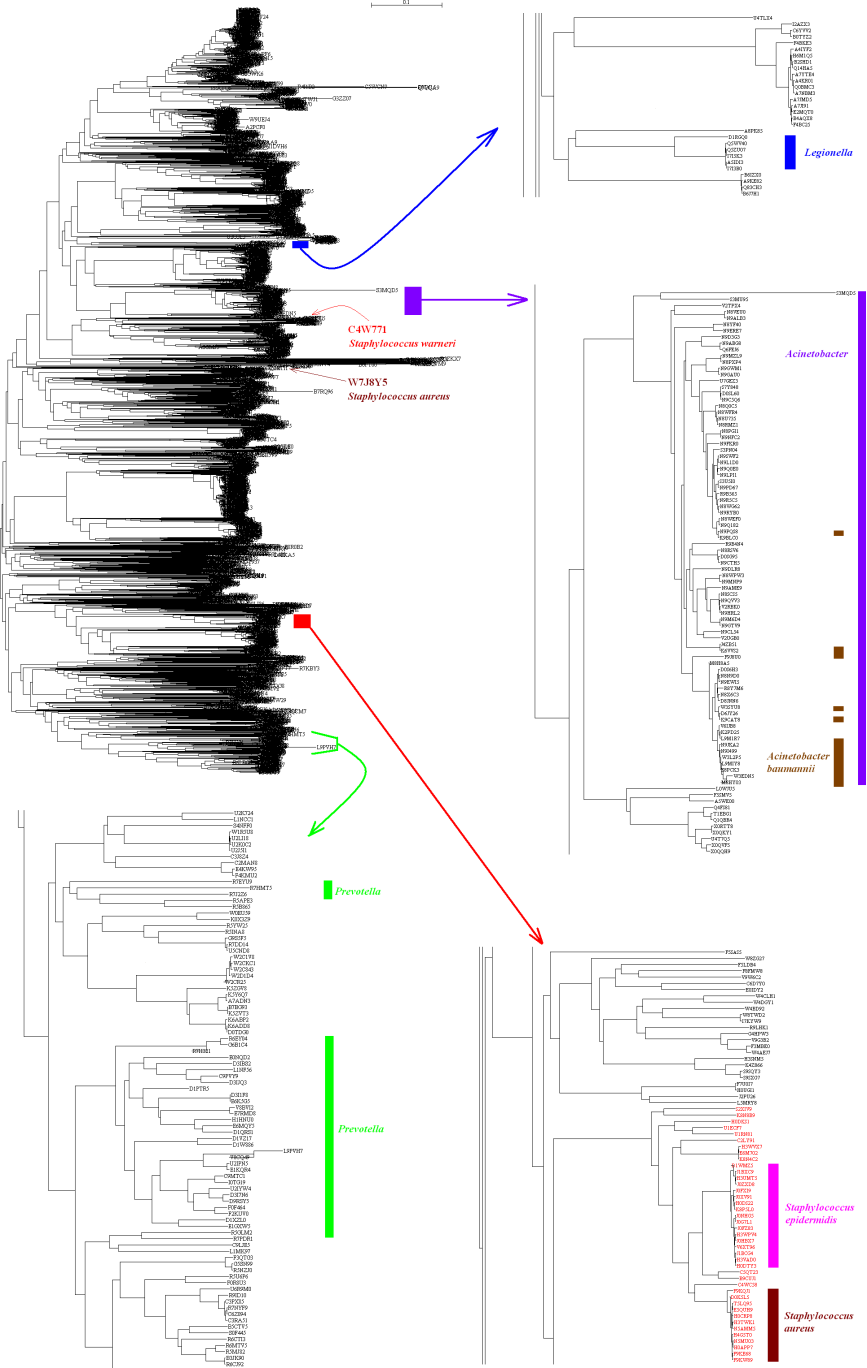
Phylogenetic tree of SecDs from bacteria. Distribution of genera *Acinetobacter*,* Legionella*,* Prevotella,* and *Staphylococcus* were marked with purple, blue, green, and red colors respectively.

**Figure 7:**
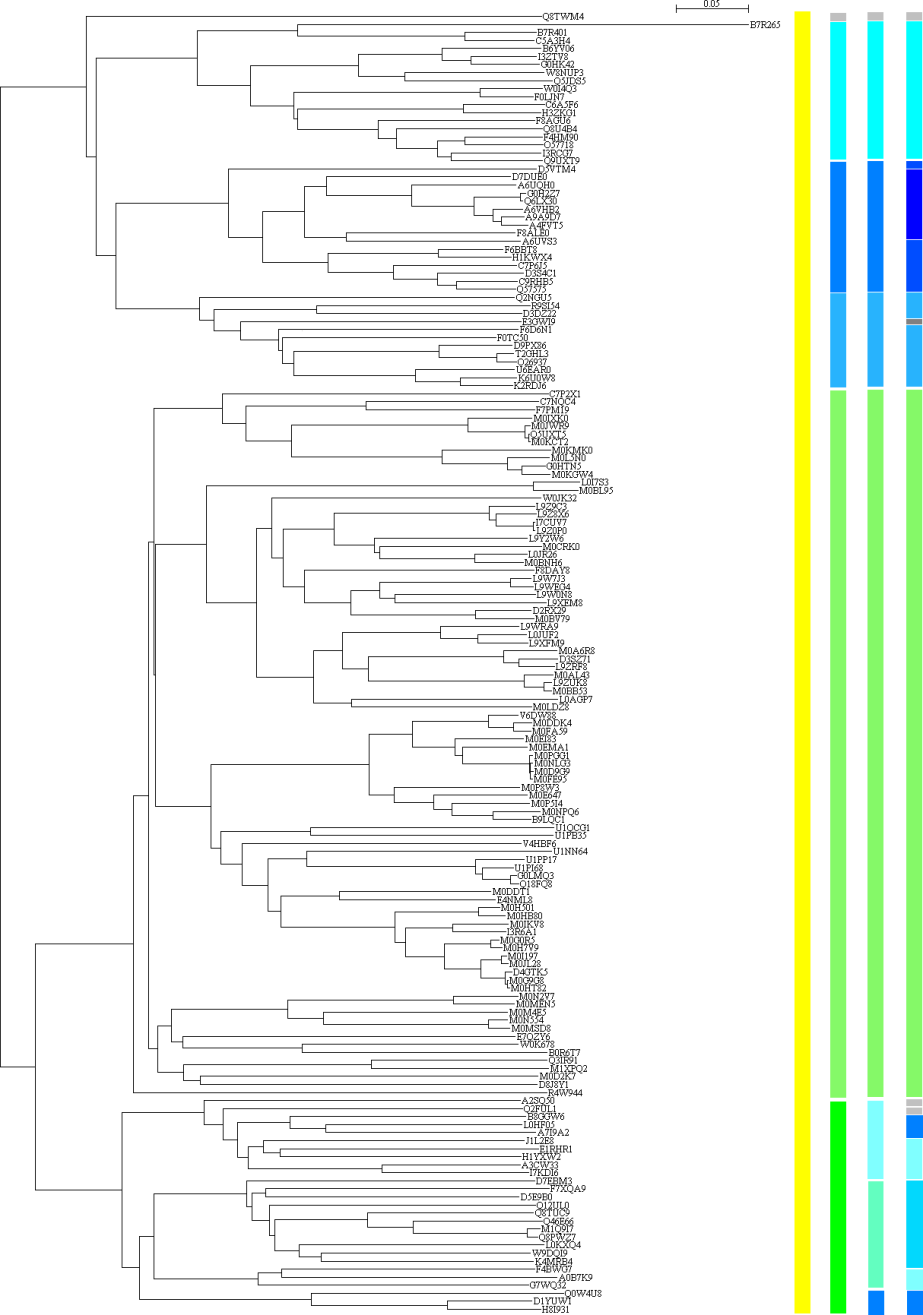
Phylogenetic tree of SecDs from archaea.

Figures 6 and 7 display phylogentic tree of SecD sequences in bacteria and archaea. As shown in the upper panel in left column of Figure 6, the sequences from phylum *Proteobacteria* distribute in the upper half of the tree while those from other phyla locate at the lower part, including phyla *Bacteroidetes* and *Firmicutes*. In general, the sequences from the four genera *Acinetobacter*,* Legionella*,* Prevotella,* and *Staphylococcus* distribute closely and form their own cluster. However, it is intriguing to note that two sequences from genus *Staphylococcus* are located far away from the cluster of *Staphylococcus*. One sequence from *Staphylococcus warneri* (accession nimber C4W771) and another one from *S. aureus* (accession nimber W7J8Y5) are located among the sequences mixed with those from phyla *Bacteroidetes* and *Proteobacteria*, which could be an example of horizontal gene transfer.

## Discussion

Dysfunction of Sec secretion system after deletion of Sec units was found to be strain dependent (Flower et al. 2000). By analogy, antibacterial agents targeting SecD would be strain dependent, so the analyses along taxonomic lineage of *S. aureus* are necessary and meaningful to define specificity of antibacterial agents against SecD.

Of staphylococcal species, *S. aureus* is the most virulent leading skin, bone, lung, and blood infections and is remarkable for the development of drug resistance; *S*. *epidermidis* causes nosocomial infections and biofilm‐associated foreign body infections; *S. fleurettii* is one of the oldest staphylococcal species where a homolog of multidrug resistant gene mecA was found (Tsubakishita et al. 2010). Unlike other staphylococcal species, no sequence of *S. fleurettii* was found in the current dataset otherwise we can trace its evolution. *S. carnosus* is a nonpathogenic species without any insertion sequences (Rosenstein et al. 2009). There is no reason to assume that microorganisms do not have many pathogen and multidrug resistant genes (Seputiene et al. 2012), for example, *Macrococcus caseolyticus* is a bacterial species that shares a common ancestor with staphylococcal species (Baba et al. 2009). In such sharing, a part of pbp4 genes was acquired through horizontal gene transfer whereas pbps1–3 was vertically transmitted from the common ancestor of macrococcal and staphylococcal species (Hiramatsu et al. 2013). *Prevotella* exists mainly in the rumen (van Gylswyk 1990) and is one of gut microbiota playing an important role on pathogenesis of various diseases (Wu et al. 2013). *Acinetobacter baumannii*, which replicates extremely fast, has many horizontal gene transfers and led a big death rate in US army in Iraq (O'Shea 2012).

As a matter of fact, the average pairwise *p*‐distance in each taxonomic group mainly concerns with the variance in each taxonomic group, which would be related to the gene flow within a taxonomic group. For example, methicillin‐resistant *S. aureus* acquired mecA gene by intraspecies gene transfer (Baba et al. 2002). Typically, this variance is not appropriate to estimate the horizontal gene transfer, which is particularly notable in prokaryotic cells (Jain et al. 1999). In order to stratify the variance of *p*‐distance in SecD in wake of the variance within a taxonomic group and the variance between taxonomic groups, i.e., inter‐ and intragroup variances, model II ANOVA is employed. The larger is the intragroup variance, the larger is the tendency that evolution is confined within a taxonomic group, whereas the larger is the intergroup variance, the larger is the tendency that evolution is more likely to go across a taxonomic boundary line. Collectively, the SecD from *Staphylococcus* species is a favorable target for developing antibacterial agents against multidrug resistance of *S. aureus* because the genus *staphylococcus* revealed the smallest interspecies variance among the four genera (Table 1).

On the one hand, *S. aureus* not only acquired mecA gene through intraspecies gene transfer (Baba et al. 2002), but also spreads pathogens from humans to poultry (Lowder et al. 2009) and pigs (Price et al. 2012). On the other hand, multidrug resistant genes were found to maintain within animals and humans separately and their transmission is limited toward either direction (Mather et al. 2013). When a species intensively entangles in a phylogenetic tree whose branches are interspersed and interwoven each other, it implies a high chance of horizontal gene transfer and can compromise specificity of antibacterial agents. It was found that *S. aureus* genome contains copies of inserted sequences and transposons, but the number of copies is limited, and many copies are inactive due to mutations and deletions as well (Mather et al. 2013). Therefore, *S. aureus* has a more conserved chromosome structure than *S. epidermidis* and *S. haemolyticus* (Watanabe et al. 2007; Jamaluddin et al. 2008). Phylogenetic analysis revealed that *S. aureus* clustered together in lower panel right column in Figure 6, except for a single one (accession number W7J8Y5) that was located far away, which could be a typical example of efficient horizontal gene transfer presumably via bacteriophages (Chambers 2001). However, *S*. *epidermidis* aggregated more closely without any exception (lower panel in right column in Fig. 6). At this point, our results are in good agreement with a study (Monk et al. 2012), which developed new techniques to make strong restriction barrier in *S. aureus* and *S. epidermidis* penetrable because the barrier in *S. aureus* and *S. epidermidis* prevents them from taking foreign DNA (Waldron and Lindsay 2006; Veiga and Pinho 2009; Corvaglia et al. 2010).

Another spotlight is *Prevotella* because staphylococcal species evolved together with mammalians as the colonizers of diverse mammalian animals (Hiramatsu et al. 2013), so it was hypothesized that the descendants of *Staphylococci* are methicillin‐susceptible, because they became protected from the threat of ß‐lactam‐producing fungi or *Actinobacteria* by the immune system of mammalian hosts (Hiramatsu et al. 2013) and mecC was found in the chromosome of *S. xylosus* (Harrison et al. 2013) while mecB has yet to be found in staphylococcal species. A recent study demonstrated that multidrug resistant *Acinetobacter baumannii* had different resistance genes through the plasmid mediated resistance mechanisms (Saranathan et al. 2014).

It was shown that some Sec components, which included SecY/Sec61*α*, SecE/Sec61*γ*, SecG/Sec61*β*, Ffh/SRP54, and FtsY/SRP receptor‐*α*, were evolved so far through vertical transmission without loss and almost without gene duplication (Cao and Saier 2003), and with some exception in prokaryotes (Bensing and Sullam 2002). The phylogenetic tree in this study reached the same conclusion with respect to SecD.

SecD sequences from genera *Legionella* and *Acinetobacter* have a far smaller average pairwise *p*‐distance (Fig. 2C; Table 1), and they seem to be specific as a target of antibacterial agents. However, both of them belong to phylum *Proteobacteria*, class *Gammaproteobacteria*, which is a large taxonomic group including 15 orders, 32 families, and 110 genera. On the contrary, *S. aureus* belongs to phylum *Firmicutes*, class *Bacilli*, which is a small taxonomic group including one order, six families, and 13 genera. A taxonomic group with fewer variants would have less horizontal gene transfer (Sokal and Rohlf 1995), so a target in a taxonomic group with small variance between taxonomic groups would be more specific. Thus, SecD of *S. aureus* is more suitable to serve as a target of antibacterial agents.

In this study, three methods were used to analyze 2686 SecDs with full taxonomic information from kingdom to species. The first method showed 0.0051 average *p*‐distance in *S. aureus*, i.e., 0.5% difference would be expected between any two SecD sequences in *S. aureus*. The average length of SecD in *S. aureus* is 741 amino acids, i.e., about four different amino acids (741 × 0.5%) would be expected between any two SecD sequences. The second method indicated that interspecies and intraspecies variances were 43.71% and 56.29% in *Staphylococcus* SecD sequences, which has an average length of 718 amino acids with 0.1562 average *p*‐distance, thus any two *Staphylococcus* SecD sequences from different species would be expected to have 49 different amino acids (718 × 0.1562 × 0.4371) and any two *Staphylococcus* SecD sequences from same species would be expected to have 63 different amino acids (718 × 0.1562 × 0.5629). The third method found that only one SecD sequence from *S. aureus* was located far away from its sister cluster, and such a separation occurred long time ago and had undergone many steps of mutations. So it is unlikely that such significant separation would occur in the foreseeing future. Taken together, these three approaches provide evidence that SecD in *S. aureus* can serve as a target for new generation of antibacterial agents because it has a small chance of mutating to become insensitive to antibacterial agents.

It is too earlier to conclude whether a gene will mutate under drug pressure after analyzing the deep history of a particular bacterial gene, not only because such drug has yet to develop but also because it is yet to know any natural products that target this gene. In reality, *S. aureus* is the target of so many antibacterial drugs, by which its multidrug resistance develops, and then there are reasons to say that each of its genes could evolve differently from the species of the same genus. However, the general evolutionary theory does show the evolution at unequal speeds with respect to each gene, that is to say, some genes are more conserved while others are more venerable, especially for proteins (Breen et al. 2012). Moreover, the Darwinian evolution already highlights different variants in each taxonomic group (Darwin 1872) suggesting the unequal speed evolution in each taxonomic group, in other words, in each environment. For *S. aureus*, its natural competence is low (Morikawa et al. 2012) so it would have a small variance with respect to each of its components. For *Listeria monocytogenes* or *Lactococcus lactis*, they seem to lack natural competence at all (Borezée et al. 2000; Claverys and Martin 2003; Wydau et al. 2006), thus we would not worry the horizontal gene transfer in them, but it is necessary to analyze the evolutionary history in *S. aureus* for the sake of possibly horizontal gene transfer affecting Sec system. Consequently, our study ends up showing that SecD is less subject to mutations in its evolutionary history. In nature, our study is only attempting to clarify the hypothesis that SecD could serve as a target for developing new generation of antibacterial agents (Quiblier et al. 2011, 2013) from the point of view of evolutionary history because this proposal should be analyzed from every possible aspect. At this stage, this hypothesis is just being put into further scrutiny, and we would expect to see additional data from various experiments to deal with this hypothesis.

Ideally, it would be preferable to conduct similar analysis on all subunits of Sec system in order to reach a convincing conclusion. However, the rationale for designing SecD as a target for the development of new generation of drugs is so strong (see paragraphs 4, 5, and 6 in Introduction) that it is more cost‐effective to conduct an analysis on SecD at first, and consider other possible candidates and then study them one by one (Yan and Wu 2015). It is true that the variation over the entire length of proteins is much less important than the variation over critical domains and/or active sites. However, such domain and active sites have yet to be defined in SecD, therefore the analysis on their variation is impossible at this moment.

## Conflict of Interest

None declared.

## Supporting information


**Table S1.** 2686 SecD sequences were used in this study.
**Table S2.** Taxonomic classification of 162 SecD sequences from archaea used in this study.
**Table S3.** Taxonomic classification of 2524 SecD sequences from bacteria used in this study.
