# Supplementary material for: Evolutionary evidence on suitability of SecD as a target for development of antibacterial agents against Staphylococcus aureus
Source: Ecol Evol. 2016 Feb 3;6(5):1393–410. doi: 10.1002/ece3.1951 (PMC4775529; doi:10.1002/ece3.1951)
Supplement: Supplementary file 1 — Table S1. 2686 SecD sequences were used in this study. Table S2. Taxonomic classification of 162 SecD sequences from archaea used in this study. Table S3. Taxonomic classification of 2524 SecD sequences from bacteria used in this study. [file ECE3-6-1393-s001.doc]

Table S1. 2686 SecD sequences were used in this study

| V4HBF6, E7QZY6, D8J8Y1, M0KGW4, M0KMK0, M0KCT2, G0HTN5, M0L5N0, Q5UXT5, M0JWR9, M0IXK0, B0R6T7, W0K678, M0LDZ8, M0M4E5, M0MSD8, M0MEN5, M0N2V7, M0N554, M0HT82, M0JL28, M0HB80, M0H7V9, M0H501, I3R6A1, M0IKV8, M0G0R5, M0G9G8, M0I197, D4GTK5, E4NML8, C7P2X1, U1PB35, U1QCG1, F8DAY8, U1NN64, Q18FQ8, G0LMQ3, U1PI68, U1PP17, F7PM19, C7NQC4, M0P8W3, M0PGG1, M0EI83, M0EMA1, M0FE95, M0FA59, M0P5I4, B9LQC1, M0NPQ6, M0NLG3, M0E647, V6DW88, M0DDK4, M0D9G9, M0DDT1, M0D2K7, W0JK32, M0CRK0, M0BV79, M0BNH6, D2RX29, M0BL95, L0I7S3, M0BB53, M0AL43, M0A6R8, L9ZRF8, D3SZ71, L9ZUK8, L9Z9C3, L9Z0P0, L9Z8X6, L0JR26, I7CUV7, L9Y2W6, L0AGP7, L9XFM9, L9WRA9, L0JUF2, L9XEM8, M1XPQ2, Q3IR91, L9WEG4, L9W7J3, L9W0N8, R4W944, K2RDJ6, F6D6N1, F0TC50, K6U0W8, U6EAR0, D3DZ22, R9SI54, Q2NGU5, D9PX86, O26937, T2GHL3, E3GWI9, C7P6J5, D5VTM4, Q57575, D3S4C1, C9RHB5, H1KWX4, F6BBT8, A6UVS3, A4FVT5, A9A9D7, A6VHB2, Q6LX30, G0H2Z7, A6UQH0, D7DUE0, F8ALE0, H8I931, D1YUW1, Q0W4U8, A2SQ50, I7KDI6, A3CW33, J1L2E8, H1YXW2, E1RHR1, A7I9A2, L0HF05, B8GGW6, Q2FUL1, F4BWG7, G7WQ32, A0B7K9, Q12UL0, D7EBM3, D5E9B0, K4MRB4, W9DQI9, L0KXQ4, F7XQA9, Q8TUC9, Q46E66, Q8PWZ7, M1Q9I7, Q8TWM4, Q9UXT9, Q8U4B4, O57718, F4HM90, I3RCG7, F8AGU6, F0LJN7, C5A3H4, Q5JDS5, H3ZKG1, W8NUP3, B6YV06, C6A5F6, G0HK42, B7R401, B7R265, I3ZTV8, W0I4Q3, , Q029K4, R5UDC9, I9EGD2, I8V2Z0, E2NDV6, R6LBG6, F3PJ48, R6CJ92, B3JK90, S0F445, F3ZR90, C3RA51, E5X114, R5JP53, K5DDT5, C9KRA7, F3PW20, E4W1D5, I9G8N6, I3HQC4, K1G4G5, K1G4B7, K1FEP9, R7DSR1, B3C7S0, U6R9M0, R9ID10, I9GBL1, K9DRU2, A7LW80, R6J3J3, I9HB64, I9IEZ1, D4W9U1, B5CTV5, U2BV10, W4PPG9, W4P9H5, W4USL5, F0R8U3, I9HYW3, C6IJK8, D1JVB4, D0TKY1, D0TDG0, C3R016, E5V7B2, C6Z094, C3PX85, R5MJ82, R5YW25, R5JV96, R6MTV5, R6CTI3, R5C7S6, R6FAD7, R5SQG3, R5U6P6, R5B865, E5CBP2, D2EXS1, U6RWK7, B0NRN3, A7V8T3, R7EJ74, I9U473, R9I3N3, R7NYF9, I9UUW9, K0X3Z9, W0EU59, U5CND8, R5UZB7, H1DJA3, R5PKY4, F9Z9H4, R6F2D5, K6ABP2, K6ADD8, K5ZGV8, K5ZVT3, B7BG93, A7ADN3, K5Y6Q7, F4KMU2, E4KW95, S4NFF0, L1NCC1, C3J8Z4, U2J5I1, U2LI18, U2K0C2, W1R5U8, U2K724, C2MAN8, G9S5F5, R5INA8, R7DD14, W2CR25, W2C1V8, W2C843, W2CKC1, W2D1D4, L1MK97, C9LJE5, R5NZJ0, G5SN99, F3QTG3, E1GXW5, D1PTR5, D1XZL0, E6K5G5, D3I1F8, D1W886, D1PDU1, F2KUV0, E1KQR4, U2IPN5, H1HNU0, E0NQD2, D9RSY5, D3I7N6, F0F464, V8CQ49, L9PVH7, E7RMD8, V8BVI2, D1QRS1, L1NF56, E6MQY5, R5APE3, R7HMT5, R7EYU9, R6EY04, R7PDR1, R5GLM2, R7J2Z6, U2IYW4, D3IBS2, I0TG19, D3IJQ3, C9PVY9, R7H321, G6B1C4, D1VZ17, C9MTC1, B0MT01, E4M8N2, G0J3R7, S7VEK4, M7XRN8, C6VU35, I2ETV3, I0K6B8, I2GRF3, E4RRE3, U5C1T7, F8ED18, D2QGW9, L8JUF1, U3QCM7, F2IIN5, K1LRH9, K1M7V2, G2ED57, F9YUB0, C2M6V2, S2VN44, C7M4E4, E4MR47, L1PMM8, L1PCS3, L1NXQ0, S3BZL8, L1P1R7, U2AXH2, E2N1H4, F0RGC4, D7W5U6, W4T3Z6, T0NG00, V6SN63, V6SD06, M7N4B8, F4B306, F6GJU9, G2PSS7, K1HSP4, H1GN25, S3YSY5, H1GV68, K1IK40, H1H5M0, J9R7Z0, L7TVR7, F0P0M1, S7VKJ8, G0LCF8, F4L4X1, R9GNF4, H1YH85, C6XT73, F0SAH8, U2HWL5, F4C3Q7, C2FY60, D7VPD6, I0GI61, Q3ANT9, Q0YQK3, B3EDP0, B3EJI9, A4SC30, B3QTH7, Q3B6Y2, B4SAK3, B4S946, E8N5I8, I0HYY0, Q3Z9L9, V5QAY1, D3SHJ4, D2BGI6, D6TLT7, Q1IYZ9, E8U9G4, F0RNU9, D7CWV7, F2NP22, D7BF10, B7AA87, G8N834, W2U544, F6DFP1, W4QU09, J8Q489, U1TCA7, V9RGU4, F4EU83, W8IN94, H8XED2, W7HB95, W4RK34, E6TV81, J7XLK4, R8TQH9, R8HGI0, J8MGY9, S3ILR3, R8E156, J8S190, J8SFW1, R8V1G2, J7V5P0, J8AF73, J8AWX9, J8BV49, J9AP72, J7XXC1, R8SSR6, J9ABN1, J9C572, J8CUH5, R8D5V0, R8CYT2, J8E4X5, R8RL16, J8F758, R8LHZ4, J8FIV2, J8GDN3, A7GT93, R8YSF4, J8DNH9, J8IP11, J8HYU5, J8IZG5, J8G716, J8KT14, R8DPX6, R8NVH5, R8SDT0, J7WK03, R8MP26, J8HQF7, R8TGZ5, R8GTB6, J8NAC0, R8Q7P0, R8EPB9, J8NG38, R8NYM4, Q5WHQ5, F7Z5M0, G2TM13, Q9KDH7, W4QGK0, U5LDV4, T5HQK5, W7RYY8, I8J0M6, U6STI3, W8QP49, G9QQ26, G9Q1A8, V7QD52, U1ZAK0, W7YUA9, X0RQ15, L8ASQ9, W9U4D6, A0RJ19, F5LAR2, D7D2U5, C5D516, E3IG00, E8T117, T0P271, B4BQ66, S5ZR75, V6VEX3, S7TZ75, B1HV93, W7RNW2, T0JGG1, D7UZ12, H1GDU7, W6AFI4, S5LSV2, F3RFQ8, U1UQT6, W6DPR9, L5MRY8, H0UGI1, F7U0I7, J2PU26, S9SXG7, K4Z866, S9SQY3, R9LHK1, E0IDY2, H3SNM5, G4HPW3, V9W6C2, F8FMW8, I7KYW9, W8TWD2, C6D7Y0, W4BD92, W4DGY1, W4AEJ7, W4CLH1, F3MBE0, F5LDB4, V9G3B2, W8ZG27, S2XQK0, W3ACZ2, F2F3G6, D0K5L5, E5QUH9, N5AMM5, N5MU03, T5LQ95, H0APP7, F9KE68, F9KQJ1, F9KW89, H0CRP8, H3TWK1, H4G5T0, W7J8Y5, B9CUJ1, C5QT23, H0DTY3, V6XT96, K8P5L0, J1BXC9, J0HBX7, J0G7L1, J1BCG4, J0ZXD8, J0FXI9, J0FZ83, J0NHG5, J0XV91, D1WMZ5, H3UMT5, H0DS22, H3VAD0, H3WPV4, U1RN81, C2LY91, K8N4C2, E6M702, H3WVX7, H0DKS1, K8N8B9, U7PAL7, U1ECF7, S2XIV9, C4WC58, C4W771, F5SAS5, A6TQN4, R8W5B7, I7KAC3, F9VKH3, G2IC17, G2IHK5, F0KAM4, K0AZP6, U5RVW7, A6LTN2, B2V343, B2TMZ7, D5VVL4, C3KTC5, E8ZS12, A5I6E4, C1FKF4, B1L0A5, B1IME6, F4A6A4, C5VTC8, C5UYU7, B1QAP4, R0AKH0, W1UBE3, D6MDU4, C6PXQ5, L1QDZ6, D9SMV2, G8LWQ4, N9WGE0, N9W3C4, D3AHT3, C0BWM2, C0BY31, U2NLX6, A5N1Z9, A7VVT8, B6FSW9, B6FT00, A0PZW1, U4R5U3, F1THB3, L7EPE5, Q8XJ19, Q0TP19, Q0SRN9, B1R312, B1RL82, U5MW29, D9R4P5, M1MY49, R9BUI2, B0NCG2, U2DCE9, V9HDK4, N1ZAN3, N2AF49, T0NAI0, H2JHT2, R5A9C8, R5P708, R5MAV6, R6BPW3, R6G434, R6D500, R5KHX9, R7ITT4, R7IWY4, R7L6W6, R6PPT1, R6N895, R6WPT7, R7F8L9, R6Z927, R6STG1, R6YDN3, R7GHL2, R7KFM9, R6Z9L6, R5ZQH8, R7BB23, R6QKY8, R6B8D7, R5ZKX7, R6I0L4, R5EKS9, R5DKZ9, R7LJH0, R5TIZ8, R7HZA0, R5PTW9, R5N5G0, R6WVL2, R7ME32, R7M2P3, R6UHJ8, R5KND2, F0Z581, E4LP13, U2BFK9, A7VDE1, D4C911, J0N263, B0P092, G9EWM0, U2B7C6, U2DBR0, E9SPB6, E9SKI0, S0FJE0, Q892A4, U6F067, A3DDV9, E6UPY4, W6N3P4, G8TXS7, E6SLR2, K6Q3K5, Q67LL8, H6LC09, V1CL21, E3GFZ5, N2AC97, U2QXE8, C7GZ32, R6RDG5, B0MK57, S0IX45, H1BL82, R5ZIJ5, R5LBT1, R6P5E4, R5NNK0, A5Z3V3, E6MEH6, B0TF81, B0MBW9, C9LBD5, U2CW28, A7B746, A7B765, V8BND7, V8BNC1, A5ZNJ2, A5KQ38, D4RYS1, V2Y925, F2JSC5, F2JHM6, C0B975, R6LPJ0, A8SRY2, R5W215, R5JYR6, S2ZQR8, B0G3M5, R7FQM0, A6BHZ4, R9MRA9, R6W8U2, E6LQG8, C6LAA6, C2KU82, D4CP12, U2WDQ9, F5T9M2, C7G8U5, C0FXA3, R7QTC0, C4G9S4, H1HVJ7, B1I4K5, K4L061, I4ABJ7, B8FQU8, G9XKW0, W0EE41, I4D995, J7IMD1, G2FKK3, G2FPV8, C8W4E1, F6B453, R4KHX4, F6DN30, A5D3G9, F0SZM2, F0T262, D5XEJ6, E8RKA1, T4BD10, B0PDU6, R9LT11, R5FHX1, R7IJN7, D4L8S6, R5TUC0, B5CSD8, R7DAG8, C6J8B2, W0U2J2, R5DY96, R5VLL5, R7FHM5, R7KPR0, R6VUH2, R6TC88, G9RYD7, C0GFY5, Q0AX22, D7CLE7, E3DQE0, M5EG95, D9QVJ1, L0K9S7, B2A263, C9R7V3, B7R9G1, Q8RAM4, Q3ABZ0, Q2RHU6, F4LTJ8, C7IUL2, F1ZUE6, D3T8K4, D7ANM7, M8CNC9, B9MPI8, E4S9Y4, D9TIR7, E4Q4C2, A4XIR1, W9EEX1, D9TNU7, L0IJY1, F6BGJ3, D9RXQ2, F4A277, B5Y8Y2, M1E629, D2MQN9, B9YCU2, E7MMN1, D2RLC8, G4Q520, R6N2E1, U2UFK4, R7LUY9, R7KBY3, C0WEF7, R6IAM1, R6WU47, E8LGB1, F7NND6, G9YFF6, F5RLQ7, R5SKG8, C9LLG7, F2BYB8, E4L7T4, R7CSE5, R6AFQ7, R7PRV4, H1D2V0, R6MDU3, R7MZ88, D3LT24, E2Z9B9, S7IYZ8, U7UEW5, F5TEW1, C9KKG8, I8RYS6, I8U5G0, E7MZ83, G5GLP1, D4S7Z7, G5H253, I0GNY2, J6IBY7, K9CI61, J4J327, E4LIU4, L1NES1, U2M065, F4EZG8, T0IHU0, A1HT86, E1LE30, C4FRS4, D1BLM4, F5L222, K9D146, J5AMK5, W3Y4B6, R5BPI3, S3ALT1, E4LG49, I6Z7F8, A6DMA1, M1YZQ2, E8R0B2, D2R231, F0SG09, D5SVB3, A6CC71, A6CFT8, Q7US98, V4Q003, F4QQ33, E8RNL9, V4NSZ7, V4Q1L2, V4P8X5, V4QMS0, U3ART3, B4WEC9, D9QLN2, Q9A6U2, R0CVI3, B0T1P6, J3AMF4, A0LCK9, J0PZT5, J0PXU4, E6YHC1, J0Q739, J1KCK5, C6ACY7, Q6G2U1, W3TXK9, J1JY96, Q6FZA3, W3TPN4, W3TYZ2, J0QHN0, E6YMK8, E6Z008, E6YV33, E6YQJ8, J0YWP8, J1K0X8, J1A2W9, J1K184, J0RFH7, J0QLB4, J0Q1P7, B2II61, B8ETD0, K8PBI2, K8PLJ2, D6V9G6, W3RDP7, Q89L13, G7D9F6, M4Z7S4, A4YV61, W1JWG4, U1H681, H0S467, H0SG16, I0G789, H5YHH9, J2WLJ5, A3WYN9, F8BM81, Q213W2, Q6N5Y7, Q2IWH6, B3Q6G7, S3VU27, U7ZF04, U7YT56, N7T8J7, N7AV32, U5C7M6, N7X221, G8T2S4, N7YIP1, C4IS16, A9M5Y2, A9MAR4, U7YLJ6, C9TEV5, D1FF64, C0G755, C0G5Z7, E0DKB6, F2GUF1, U8A480, N8JNY4, C0RJT3, C0RIM5, D1EYU6, D1F8E7, N7MTZ3, N7MSW7, N8DVZ1, N8F6Q0, C9TM61, U7XWV5, N8EDG3, N8G4K2, D1CXN5, N8GFH9, E0E0L2, B0CHB7, B0CLL7, U7WGN0, D0PIT4, D0PCL4, A6X022, A6X1E6, U4VK69, C4WEC0, U1YQM4, U1Z064, D8JWH1, N0B5R6, F8J668, G4R9T0, B7KPX9, C7CKS4, A9VY43, H1KKW3, M7YR71, B1ZJ04, B1LXR4, B0UI92, I9LJI8, I4YX25, J7QNK4, Q11HD5, E8TKC7, M5F692, F7Y4M8, V7HDZ8, V7H4H7, V7FGC2, M5FDU2, B9J938, W1L583, Q7CYZ3, W8FFL9, B9JXD2, B9K2H0, U6B5V9, L0EVK8, W8IAS1, B5ZQA8, C6B1B3, C6AWE3, B5ZMG6, W0N4R9, W0N395, W0IDA7, W0IH32, K0PG49, K0PSM3, L0NKK7, W6RCR5, H4FDP7, H4F629, G9A5T1, G9AAU4, C3MBU9, A6U8P5, A6U5W0, F6DZ25, F6BMN6, F6DY48, F6BQS7, A8ID85, D6ZZ42, A7IND6, C6XJ02, Q0BZM0, Q0C0W6, A3UDL9, E0MKQ8, K2K5T7, I1B1B5, A8LN88, Q28TG1, E3F0T8, A0NQN5, B9QZH6, U7GKU7, V9VTH8, U3ABB8, A3V9B5, A3VMR6, A9E896, A3U2N1, Q2CHT2, K2H7E6, S5XSI1, A1B1X3, Q0FH69, B6QY42, O33517, V8H759, V7EED1, C8RZF2, Q3J5I9, A4WPP8, B9KLH8, J6UEV9, W8RXZ2, W4HP29, Q166J2, F7ZKY2, A6FP29, A4ELF6, B7RQ96, A3XAG0, A4EUX1, A3SRE5, A3W5Q9, A3VXM7, A6E4F9, S9R5F1, R0F780, Q5LR11, Q1GHY7, B7QQM6, D0CXZ0, A3KA01, S9Q4J0, A3SXR4, C7DE01, C7JGJ8, S6D819, F1YQC5, R5QLM6, F7VEY8, F0J0P9, V8A971, D5QD74, F3SBL1, G2I3G5, G6XI08, Q5FNB4, K7TBS9, T1DDZ2, W6I333, W6IRC4, W5Y5T1, D5RRE8, G1Y192, G8AQR7, G7Z513, R6J8A0, R6HUK5, K7YF97, V9TUC0, M2YCR4, K2JMG4, H8FUE2, H8FR23, B6ISK0, B6ISM9, H6SQ52, Q2RTI1, W9H7L2, K2M8W6, I3TWR6, D1ATG6, Q5P9H2, U5XWB9, Q2GID6, S6G668, Q3YQU0, Q2GF93, Q5FGJ8, Q5HA34, C6V5A8, Q2GDE0, B6Y8L4, Q4EDD8, C0F9Q4, I7JAT0, U4P928, H0U3G3, U6SZB2, Q5GRJ8, F7XUZ0, B3CTC4, C3PP22, A8GP54, H8K5M2, A8GWN1, A8EY98, H6PEE2, Q92H77, C4YYX2, Q4UKW3, G0GYU0, G4KL39, A8F266, H6QIM4, H8KF42, H6PUH7, Q9ZCW8, M9TFM1, H8KKP1, B0BYD6, H6PNW0, Q7P929, G8LB81, H8LPP0, Q68WF2, A3W9F7, A5PEI4, W9BYZ5, Q2G6L1, T0HBL4, F1Z9F9, G6E7D2, J2GWZ5, I9C924, T0HVD1, I5BJ40, D4YYJ0, N1MSB8, T0IV07, J2LB53, W1SCH4, T0KJF1, K9CZJ1, W0ABL7, J8SN89, M4S044, F3X2D6, Q1N7H7, N9VYB8, F8DU75, I6YJ04, V5Q4F9, F8EU99, H0F7G9, J4YUL9, E3HRI1, F7SVZ8, E5U7K2, J0ULA2, Q7WMN0, K0MUM2, K4QKT7, M5NPS8, J7QRV1, W8X0V6, V8FXT7, F4GQ99, G4QA10, E8UEP8, I7IIP4, Q0BI54, B1YTG7, B1FPP9, Q1BZ05, B1JVY6, T0F117, J7J5A2, A2WCJ1, F2L7S5, B1FWH5, Q62H80, A9AGF1, J4R7R9, J5B0Y7, B9C0J8, K0DID1, B2JGH1, A3NDC0, A3NZ30, Q63R03, B2H0V8, S5PEV8, W1M854, C4KUK3, W9UL72, C0YB46, B1HAQ8, E5AM58, E1T7P8, Q39JF7, E8YN33, I2ICS5, B5WQZ9, I2DK18, R4WF92, K8RFM4, F0G8U2, G8M433, I5D1V5, Q2SZ26, W6B536, N0AD62, U2F9Z1, Q145I2, G2J9I0, G0EVV6, Q0K731, Q46XG2, V2ILR0, B3R6J6, Q1LJ57, E7RY59, A6GPT1, V5PV83, V5UGX8, R7X2P8, A4SZU0, B1XRW1, B2UC77, R0DS66, F6FYK7, D8NYM6, Q8XVW2, D8N6U9, B5SHU3, V4ZU07, U3GIR7, S9RWQ1, R7XAJ4, I9W4G3, F0QA90, A1TW41, C5T8N7, B9MHS3, K0I6P8, F4GA72, B7X295, C9YD64, A9BS74, S2X938, S2W9A7, F6AMV4, I4MT53, F3KW70, A1VU53, Q122L4, J3D1A6, F5Y245, T1XHI1, J3CW19, A1WQ85, G0AI17, R0EDH6, J2UKB2, I3CYC8, J3CC25, A4G1Z5, W0V0U7, A6SUU6, L9PIM2, L9PPQ8, C3X617, C3XC96, R5EJI5, F3QH22, H3KCR7, R7I7E6, R7BZS7, R7KV22, K1JKI9, R5PN05, S3C6S0, D9SJA7, D5CQ35, S6B677, Q3SH57, Q1H405, D7DM25, E4QLB4, Q7NYC9, C0DYW4, V7IAG9, F0F2V0, F5S527, C4GGK5, C1DDA9, D0W1X9, C0EJI5, Q5FA43, B4RJK8, C1HVU4, D1EDM4, E4ZEP5, D0WBP0, E1P2Q6, F9ETM2, C6S5M7, R0X814, L5UV80, T0XAZ5, L5QSE6, R0X9A3, L5SXX0, L5TY20, R0VFA6, L5V1D0, J8XP01, J8W956, J8XPP3, C6SDK9, C6SDL0, I7L1G1, E0NAI2, E9ZVX7, T0W8W7, J8YRV3, A1IQM3, Q9K0J0, F0NAI9, C9X0W1, A9M2E8, D2ZSN5, E2PHL4, G4CGP3, C6M8Z0, I2NSM2, D7N3K1, L1NKU0, G4CP72, G2DQU6, B9Z6Y5, U0ZBZ6, V9HMD5, Q0AG53, F8GIE1, F9ZGF1, Q2Y6A5, M5DL92, Q5P718, A1K3W6, G8QJX6, Q47AX1, F5R7F7, W0SGT4, N6YUK0, N6Y7L8, N6ZR83, C4KCQ5, E1X0T6, T0CE01, T0STU2, Q6MKZ2, K7ZFV6, M4V8C2, I5B224, K0NFC9, S7U5Y3, A8ZW12, S0G1H5, Q6AQ38, D6Z188, C8X140, C7LN34, G1V4K3, E5Y937, E5Y454, E6VWP3, F3Z2W2, S7T1E4, B8J2Q6, F0JFM1, L0RC11, C4XSM5, M1WQJ7, B6WR37, D9YG74, G2H4D5, I2Q0M4, Q72B20, B8DKR9, Q1MSC1, W0JK09, F2LVF9, Q1K1D7, B5ECA4, B5EFX6, Q39XC8, C6E6J7, Q749X5, Q3A3C4, A1APZ0, S9QT49, Q08P79, D0LJ86, B8JH52, Q2IKX9, B4UID6, A7H9Y5, H8MU10, F8C740, U2RT80, L7UEH7, A6G9V8, A9F8D1, S4Y111, F2NJP6, I4CD43, I4CFA0, Q2LVH4, A0LHM9, A8ERU6, S5PSE4, G2HL64, E6L219, D5V284, G2HTF0, H7QT53, H7T3L9, H7THJ3, H7TAH8, H7VSX4, H7U1Z6, H7U8V9, H7RHM4, H7RSM1, H7RUR7, H7S865, H7UZE9, T2LIU9, H7SP11, H7QZ25, H7WQ26, H7WAT4, W6MZZ7, E0QCF6, V7ZU01, H7W3N5, Q4HGN3, W8KNC3, A7ZE42, U2FDV1, U2GES0, U2EKI2, U2GWR2, I1DNS3, A7GZ84, A0RNX0, C8PJX8, A7I0M3, Q5HU11, S5IZS7, U4P9V8, A7H2R4, D3FMU8, H7X124, D2MTK4, H8CB07, H8CKL6, H8A201, H8A9W2, H8A4I8, H7ZTV1, A3YP46, D2MYL6, H7XUC3, A3YMG8, A5KFI1, H8D0S1, H7XKQ4, H7Z1R2, G8F706, A8FME6, B9D3N9, M5IPF8, C6RD36, U5XYG2, J5KTN5, S3XJF1, I3XYF2, D1B3G7, W6EJD3, Q17YZ3, F8KRR3, H5V9P8, C5ZWZ2, V8CLC1, I0EKD5, I0ES72, I2FC74, E7A9U3, T1CZ05, K4RHG9, Q7VFM8, V8CCM5, D3UIE1, C5F1Q6, Q9ZJ66, E1PYB8, E8QUX0, E6S3R6, D0K1M9, K4NHF4, C7C1C3, D7FGC8, E1QA59, E6NFZ2, E6NTT3, Q1CR56, E8QEW8, B6JP52, E1PUF9, B2UVW0, D6XS58, B5Z9I1, F2JG55, F4D4L7, L8VUS2, K7Z6R2, M7STZ3, M7SPT7, I9XXZ0, I9P8A6, J0I844, T5C0U3, T5C8J7, T5CSF6, T5CNV3, T5D7V7, K8GVH5, M3L2M4, M3L308, M3LSK6, M3LBW1, M3N0N2, T9XAC9, M3LXT7, M3NG75, M3M9F8, M3NPN8, M3LNY1, M3MRG3, M3MWA3, M3N186, M3PDJ7, M3PSB7, M3LZP3, M3QG51, M3QZN2, M3N655, M3R8T5, M3Q013, M3QV20, M3QAH1, M3QFF1, M3SBQ8, M3SCZ4, M5YN48, M5Z0T6, T5DJT4, J0CDY7, J0MS84, I9RX44, J0CNL8, J0JZY7, J0K0T3, J0M9F2, J0C4H3, J0NM82, J0KGZ0, J0P1M5, I9VAY8, I9VDU5, J0PAQ6, J0KQF8, J0AUK7, J0L0W3, I9SNZ2, J0N7K9, J0L768, I9W156, J0BIK2, J0N3S9, J0BE66, J0LK35, J0LZD1, J0LUB8, I9YJV9, I9URB4, J0EI58, J0F0Q8, J0FDM1, J0PL84, J0QVW9, I9YVF5, J0AA68, J0SIS9, J0PWI1, J0TVX1, J0PYF4, J0AGI7, J0RME4, I9XN58, J0EZ12, M3ULH8, I0EZ09, J0J349, I9QEU9, I9QTZ1, J0IPE1, I9PVR6, J0IZT1, M4ZLC9, M4ZQS8, K2JI12, K2JKS4, K2KUM2, K2KY43, K2K0B2, K2LKH3, K2LMM9, K2KXQ3, I0EFP4, I0EB81, I0E6V6, T1UDA7, T2SDX7, T0DBY5, R4QGJ6, T0DRR0, R4QMT8, U4RKA4, T0E0I8, T0G6E0, S5NJB2, M7SEA7, V6LJE9, I2DI20, E7G461, C3XP01, E4U018, K7S828, Q30Q79, B6BKW4, H1FW57, T0JFP7, J0LAK7, Q7M924, A6DD49, B9L684, G0JN86, B5EK06, F8XMP4, N9TZE1, N9TXV9, V9ZTV7, R4VFB4, R4VEN7, K1JSL7, K1JP99, A0KJ22, A0KLF1, R1F5Y1, R1GTP6, A4SLM7, G7CPL4, F4DDU1, F4DGN2, K1IKK4, K1JJB1, K1JLX5, C4L7L1, E8LK28, R5EKW5, R9PJE2, R9PPA3, I9DRS1, H3ZGM6, F2GAW6, K0D388, F2G8L5, K0CUX0, S5B120, S5AH65, F5ZAV2, K6W4V1, K6YXS9, K6YUV9, K6XW98, K6ZKQ0, K6ZD47, G4QK27, K6Y506, K7AC96, K7AAA9, H5T7W2, E4PKE8, A6EWH5, A1TZN9, H8W738, G6YV88, N6WYD1, M7CSR9, M1FEU8, U7NT86, A3JJN6, A3JAG2, U7G409, Q21KW0, Q487A0, E1SV05, F7RYA2, A6F9M8, A6F8V6, N6V252, U1J872, U1JEG6, U1JFE8, Q3ILB6, Q3IL52, F3BE11, F3BPF6, V4HCM3, L8CZ90, U1L2C3, U1K3Q8, U1L9K2, U1LVE3, E6RI45, G7EX13, G7F8K3, G7F302, G7FFA8, G7FGH9, G7FTH4, G7G7P6, G7FY32, G7ECG7, M5GZM3, W1Z8T7, W1YXG0, U1L8Z9, A4CAW8, A4C7I5, U1MN93, A1SWT8, M4U5V1, A1S7P0, A1S8J5, A3D6A9, A3D7L7, A6WRI0, B8E6M1, G0ATE2, G0B0J0, G6E4E8, V1D9V0, V1DDV2, Q12PD7, Q07ZM4, Q086I3, B0TND6, A3QFF3, Q8EHM6, Q8ECM5, A8H2L0, B8CLC7, B8CKG2, E6XRC4, E6XJ97, A8FYT1, A0KTY5, Q0HLH2, Q0HXS6, A0KV52, Q0HWQ7, A1RI69, A1RGW4, F7RJW5, D4ZIP8, D4ZB76, C8N9K3, G9ZJL2, A5EWX7, W0E1R1, Q3J8N8, D8K801, I1DSN3, I1DVH6, F7P0M3, F9U703, L0GV49, G2E2L1, W9V2R1, H8Z4H7, Q0A9C0, W8KIF5, A1WXP8, U5T275, L0DY03, W0DLX9, G4E1L9, D0KW97, D2TW99, G7LNW7, Q7VQA9, E8Q615, C5WCN9, C4K8M1, G2GWK6, S3J320, G9SG40, R1FQI1, A8AK46, D2TKZ4, D4BD58, K8A0U2, K8B415, K8AWB0, A7MLX6, K8C817, V5U348, M1JHI6, C9XX71, K8BDI4, K8DHQ0, C6CBF1, U6ZHG4, C5BCF9, D0ZE75, D4F2X3, M0QAN9, Q9XBN2, G0E7G3, L8BK37, G2S989, D2ZFY5, V3IY43, V3J9L3, S9Y1W4, G8LPA9, W1FCP8, V5ARF6, S7UBR7, J7G9E6, I4ZMM7, D6DVT3, V3F7L2, V3H938, E3G706, W6J1N3, A4W781, J1GIP3, V3QVH1, V3R3U6, R9VT03, D4I7P7, V6CMA5, D8MNV3, V5Z519, D2T4N7, D0FUU6, E3DIX3, R9NPP0, W0ARJ9, C6UBM0, G7RT11, C9QQB0, E1S1J9, N2M5J9, E2WRF3, E3XHE3, M9BSF4, M9B7I3, M8YQB1, M8XV19, M8UF09, K3HXH0, V0ZYX4, V0B2Q3, K3I5D1, W1SVB5, H4HT54, H4S6R0, V6P0A7, H1F8L2, F4VAI9, V4F0T8, T6X3M9, T6Z469, T7C400, T7FFZ5, T7GW33, T7TNV0, T6MMS1, W1FUQ9, T8GN46, T8KB03, L2W5R0, S0U6Z6, L4J0Q7, S0WUE7, L3YLW3, I4JBW3, M9GQ75, M8ND76, M8LUQ7, M8MVB2, M8KYQ2, B7UJN1, N2IGD2, N4MH12, N2WF76, N3AX68, N3F1U9, N3FLD4, N4NEA4, N3H8M0, N4BPQ3, N4K1S7, N4TC53, G2ABB2, U0CFB3, T8W1Q3, E9Z4U6, H5V4K4, G9YDB7, G8WA01, H3L3N8, H3LK22, H3MIT9, I6WL27, K6L8H9, U6T9J0, V2ZJU1, W1AV24, W1B2V5, V3RMT2, M5QLI2, K4H6R8, W0XPU8, C4X4F3, W8YDF7, D6GFF6, J2IPI6, M7D306, J7U1N7, L7BRC7, U4VYA6, D4GL86, U4WG96, U1V9L2, E6W7X5, E0LTQ0, U2N1Q7, J3DFY7, H8DII2, E1SF67, Q6D853, C6DB28, K4FWR6, J8PRX8, U7R2P1, W3VBB6, R8ATQ5, V6MMX3, B4EU15, S5TXB9, K8WWV4, B6XA49, W3YCP4, K8W3B7, D4C1E4, D1P6C0, K8VZF9, I0DSD3, H8NYN7, E8XQE5, M9VZY2, B5EWU1, A9MM55, F8VNA8, S5MSF1, Q9ZFF8, V2J2U5, U6Q5I0, U6Q9S9, G5L5A5, V3VP00, N1GQK6, V2GJI7, V2DQP0, L9TPS2, J2HA63, I0NG06, G5MUC9, V1YS52, G5N8N5, G5NPU1, V1XSL6, G5P4K0, G5PJ10, G5PYX9, I9KBH8, M3L3F6, V2MU00, S3E939, H0NBX5, G5QVK7, T2K3M1, U3SGK8, M9XNH6, G5RBH4, G5S790, R0DHV8, U2LRR2, W0LGM4, S5EFR5, U1VXZ1, L0MBQ5, W0SM63, D4DWD1, G0BAL5, S0ADS2, A8GAM7, V3T737, V5BUI9, I0QNB3, R4I1B2, E9CP68, I6G7W9, E7SNH2, I6C325, F5NCB0, I6D601, Q2NVA2, W0I0S5, D3V0X1, W1IW23, N1NKE9, C4UEH7, C4RZE4, N1L7R1, N1L0V7, N1KRJ4, L0RQI5, W8TYB8, K1C1D0, F0L0U7, F4N7I7, C4SMK9, C4SWX3, C4U288, C4S7T9, A4TPH4, D0JFZ4, B0H1L3, D1TS53, B2K6S8, C4UZ70, C4UK01, W8GGX2, B6IZX0, B6J7H1, A9KE82, Q83CH3, A8PK85, D1RGQ0, A5IDI3, Q5WV40, Q5ZU07, I7I3B0, I7I5K3, G3IS61, Q60B03, V5BVE7, G4SZ44, Q0VS97, K0CGD4, K0CEA6, L0WAU6, K2FUS6, U7G1W9, U7HW78, C7RB47, Q2SDV3, S2KN61, G9E997, E1V6F4, T2L4B2, W7PYR6, W1NB75, H0J574, G4FBW5, U7NED6, F7SK52, L9UAM0, B9CXA1, C5S2U6, B0BQ04, A3N173, B3H1V0, E0E8R3, E0F4N8, E0FB22, E0FH20, D9P7A8, E0ESL9, K0G2E9, E8KG48, G3ZG74, G3Z9S3, I1XPP7, H0KHB6, G3ZZ07, G4A959, G4AH12, C6APA6, G4BEC6, E6KYW6, U1R375, W0QVY3, W0QZE1, W0R933, F4HCD9, F2C2V7, Q7VKW0, F9GI18, F9GNQ8, F9GTL1, F9H724, Q4QNU7, P44591, E4QWY6, E1X9H0, E7A846, T2BI54, A4NLL8, I3DHM2, E1W1L3, F0ES58, I3BC65, I2J9I3, I2NKA5, U4S877, T2RJG0, U4SGG7, N1VGU0, U4SUV1, R9XP31, F9QBD5, H1LMT3, J4TWJ1, Q0I4S0, B0UWK2, S5E4Q5, S5F8N2, S9Y723, M9X0Y3, W0Q7H5, W0QD70, W0QP20, I3DCM8, H8IDU0, G7SW93, S3GJD4, E8PCK3, M8H8A5, M8HY03, F9J8U0, W3SYU8, N9JKA2, N9I499, L9M1R7, W3EDN5, W3L2P5, K9CAT8, K9BLC0, L9MIY8, Q6FEJ6, N9FKR0, N9ERE7, N9DLR8, N9ALB3, R8Y7M6, N9EWI5, N8N9D0, D0S6H3, N8YF40, S3PN04, N9GAU0, N9GWM1, V2UGB0, N9CTH5, N8RSV6, D0SG95, N9C5Q6, S7Y848, D0SL60, N9GTV9, N9HRL2, V2RBK0, N8SCS5, V2TPX4, K2PD25, V6IJB8, D8JNN6, N8RMZ1, N8Q0C5, K6VVS2, J4ZB51, S3MU95, S3MQD5, N9AME9, N9ABG8, N8VEU0, N9NFC2, N9RYB0, N9LPI1, N9L1D0, N8WFR4, N8U735, R9B565, N8WG62, N9PD67, N9QVV3, N9R5C5, N8PXP4, U7GEZ3, N9SWF2, S3U5I0, N9Q0E0, N9Q182, N9MNP9, N8PGI1, N9MZL9, N9PQS8, N9M6D4, N8WEF0, N8X6C3, N8WPW3, D6JY26, R9B4N4, N9CL54, N9D3G3, C8PZC3, L0WJU5, L2F5U0, U4T7Q5, Q4FS81, Q1QBR4, A5WE00, F5SMV5, T1EBG1, X0QKY1, X0RTT8, X0QQH9, X0QVF5, C1DE74, B3PDJ8, I3I5U7, Q9HXI1, A6V0U1, U9AKT3, E2ZYS4, K1DQ80, M9RY05, U9L2L1, U8MMY3, U9J7E8, U8L2S6, U9ELA4, U9ML33, U8RJ86, T2ENW3, J7DF62, U9ED17, R9ZGU1, U3B5U8, U3HAQ7, K2SP11, F2K8D1, W8PMX2, V4QDU8, J2EUT7, W0H160, M4X271, C3K1N2, I2BRA5, V7E654, L7GVS7, G8QA86, W2F625, U7DJ17, J2Y3Q0, I4K715, E2XXS5, F6AB03, F4DUC6, A4XY50, U1SZ81, V9UDG9, V8R0N9, V6UZQ1, S2JYN2, R4RE80, W6R644, L8M866, E4R515, I7BEB0, B0KPG9, Q88PL5, B1JDS0, L1M032, R9UZ70, L0FGP7, U2UI58, M7RAW1, T2HCX3, V4GW98, V6JAG1, S6BCU0, D7HX35, W5IZL2, F0E0D3, W9T7C3, S6JLR5, S6K2I2, S6IZ36, K5YNI1, U2A0C0, V9WPK8, W2DUP1, J2MIY9, J2MMH6, J3EXZ6, J2NRR8, J3F6G9, W6VQN3, J3FHD9, J3GPG2, J2U1Z5, J2TRD9, J2XYT8, J2WRH3, J2XKA2, J3IX04, M5QH43, I4N895, K9NEU3, W8R1D8, F8H919, F2N6G1, H7EZ61, I7A256, U3HMJ4, M2V7X0, L0GJ36, I4JLV9, I4L3U5, L7GCH8, F3GVG4, F3JBD0, S6W605, S6SHE9, K2TRP9, E7P9A3, F3IQN8, F3HEK5, F3FAA2, F3DPY8, F2ZKJ4, Q48M12, V7D7T6, K6BYK8, I6LCJ7, U2G3L3, F4BKE3, F4BC25, I2AZX3, B4AQX8, E2MQT0, A7JMD5, A7JI91, B0TYZ2, C6YVV2, A7NBM3, Q0BMC3, A4KR01, A7YTE4, B2SHD1, Q14HA5, A4IYF2, H6M1Q5, S2UDW2, S5TF93, F5SXK0, F5SZX9, I1YKQ4, I1XK90, I1XL86, C0N4C2, U4TLX4, W0DR02, F6DD18, Q31FZ7, I3CL02, A7BZE3, B6EK63, Q5E3D4, Q5E6H6, B5F9Y1, B5FCH4, H1R0W5, D0ICE1, R1ITC9, R1IRW8, Q1ZRC9, D0Z389, D0YZK5, X0NXJ0, W9A223, W9A678, W9A640, Q6LU67, Q6LK07, Q1YX62, Q1Z727, L8J8U8, L8JH57, Q2C7I2, Q2C0U7, T1Y162, T1Y3M5, C2HV79, Q1VDH7, D0WXP7, M2RVJ7, M2T3R2, U3BUF7, U3A8A8, T2BZ39, E9RGS3, F7YNK7, U3ARS4, U3AVE9, E8LUY4, E8LPX5, A7N3X2, A7MU53, L8XDU2, A6ANE6, E3BML5, C2CD98, C2C913, A2P2V2, A2PCF0, A1FA42, A1F0K0, L7DYG7, A6ACS8, A6XTY1, C6S224, K5KHG1, K5M6J6, K2WE45, K2WPI9, F9BRS1, K5LU14, J1E4J6, K5NPW1, K5TKR4, L8T5A5, F9ADC0, F2ITZ8, A5ZZT7, A2PPZ9, A3GNV4, A3GMJ5, M7L734, M7KUB6, L1QT46, D0H5K1, D7HCC5, D7HET4, C2IBC1, A3EI59, A3EIM6, A1EI12, C9NU96, C9NQR9, U0G3L4, U5A8W7, U4ZY11, U3CHR2, S7HZR0, S7JXQ3, S7HUU9, F0LVW9, D0XDJ7, M7RH30, C9P778, C9P6D4, U5A3J7, U4ZLN8, M5N8W2, M5N9A0, D0GZZ8, D0GSQ8, D0HEB6, D2YHR6, D2Y9G7, S6LE31, U4DP88, U4DJE9, F9TLZ4, U4ELH7, U4GQH9, U4INK6, U4JA43, F9SWT6, C9QGS2, C9QL43, T5EGT4, V7DJS0, T5GI21, W3YQ37, W6XL24, T5IV77, W9UEJ4, W2B1L7, E1ECI7, W6DCV2, T5ENR6, U2ZJL3, F9RLI3, F9RV78, A6D752, A6CVN0, A6CVM9, E8MCY2, A7JYQ7, B8K8D0, B8K7B7, A8T799, A8SZX0, A8SZX3, H2IK40, K5VBJ9, K5VDG8, A3XVS2, A3XSG2, F9R9T7, C9Q8F4, D0IJA3, D0IGT5, B7VU10, B7VQL0, A3USV7, A3UNY6, F9SGP9, F9S9K9, F9T8F9, I1D9S6, Q7MD23, Q8DEY2, Q8D603, E8VVC4, I7ZCG2, H8L1P5, E6WUK9, M4NC76, I4VNB0, I4WGA5, I4W5F3, I4WEF5, B2FN02, W7G3D1, J7SWW4, M5TN78, I0KMR7, G0JWG9, T5KWF9, B8L1V0, L8Y209, D2UE20, W4SCB9, W4T052, G2LW33, K8FW27, M4TYT6, Q4UVX0, G0CHZ1, Q3BS39, H8FKF5, D4T1P0, D4SQF8, U4M7X9, V7ZB30, Q5GZY5, B2SIP1, G7TBP1, L7HDY8, L0SW97, F0BB47, Q9PGS3, E1RPB2, B0U1P8, V8KIM6, Q0EY70, D5EDU5, I4BUY7, D2Z8Y0, H0UIH9, D1Y6M0, D1B608, G7V8Q6, D5EKA5, R7L400, B9XN52, B2UL50, R6J9D8, R7DSW8 |
| --- |

Table S2. Taxonomic classification of 162 SecD sequences from archaea used in this study

| Phylum |  | Class |  | Order |  | Family |  | Genus |  |
| --- | --- | --- | --- | --- | --- | --- | --- | --- | --- |
| Euryarchaeota | 162 | Halobacteria | 88 | Halobacteriales | 88 | Halobacteriaceae | 88 | Candidatus Halobonum | 1 |
|  |  | Methanobacteria | 12 | Methanobacteriales | 12 | Methanobacteriaceae | 11 | Haladaptatus | 1 |
|  |  | Methanococci | 16 | Methanococcales | 16 | Methanothermaceae | 1 | Halalkalicoccus | 1 |
|  |  | Methanomicrobia | 27 | Methanocellales | 3 | Methanocaldococcaceae | 7 | Haloarcula | 8 |
|  |  | Methanopyri | 1 | Methanomicrobiales | 10 | Methanococcaceae | 9 | Halobacterium | 2 |
|  |  | Thermococci | 18 | Methanosarcinales | 14 | Methanocellaceae | 3 | Halobiforma | 1 |
|  |  |  |  | Methanopyrales | 1 | Methanocorpusculaceae | 1 | Halococcus | 5 |
|  |  |  |  | Thermococcales | 18 | Methanomicrobiaceae | 5 | Haloferax | 11 |
|  |  |  |  |  |  | Methanoregulaceae | 3 | Halogeometricum | 1 |
|  |  |  |  |  |  | Methanospirillaceae | 1 | Halomicrobium | 1 |
|  |  |  |  |  |  | Methanosaetaceae | 3 | Halonotius | 2 |
|  |  |  |  |  |  | Methanosarcinaceae | 11 | Halopiger | 1 |
|  |  |  |  |  |  | Methanopyraceae | 1 | Haloquadratum | 5 |
|  |  |  |  |  |  | Thermococcaceae | 18 | Halorhabdus | 2 |
|  |  |  |  |  |  |  |  | Halorubrum | 14 |
|  |  |  |  |  |  |  |  | Halosarcina | 1 |
|  |  |  |  |  |  |  |  | Halosimplex | 1 |
|  |  |  |  |  |  |  |  | Halostagnicola | 1 |
|  |  |  |  |  |  |  |  | Haloterrigena | 4 |
|  |  |  |  |  |  |  |  | Halovivax | 2 |
|  |  |  |  |  |  |  |  | Natrialba | 6 |
|  |  |  |  |  |  |  |  | Natrinema | 6 |
|  |  |  |  |  |  |  |  | Natronobacterium | 1 |
|  |  |  |  |  |  |  |  | Natronococcus | 3 |
|  |  |  |  |  |  |  |  | Natronolimnobius | 1 |
|  |  |  |  |  |  |  |  | Natronomonas | 2 |
|  |  |  |  |  |  |  |  | Natronorubrum | 3 |
|  |  |  |  |  |  |  |  | Salinarchaeum | 1 |
|  |  |  |  |  |  |  |  | Methanobacterium | 5 |
|  |  |  |  |  |  |  |  | Methanobrevibacter | 2 |
|  |  |  |  |  |  |  |  | Methanosphaera | 1 |
|  |  |  |  |  |  |  |  | Methanothermobacter | 3 |
|  |  |  |  |  |  |  |  | Methanothermus | 1 |
|  |  |  |  |  |  |  |  | Methanocaldococcus | 5 |
|  |  |  |  |  |  |  |  | Methanotorris | 2 |
|  |  |  |  |  |  |  |  | Methanococcus | 8 |
|  |  |  |  |  |  |  |  | Methanothermococcus | 1 |
|  |  |  |  |  |  |  |  | Methanocella | 3 |
|  |  |  |  |  |  |  |  | Methanocorpusculum | 1 |
|  |  |  |  |  |  |  |  | Methanoculleus | 2 |
|  |  |  |  |  |  |  |  | Methanofollis | 1 |
|  |  |  |  |  |  |  |  | Methanoplanus | 2 |
|  |  |  |  |  |  |  |  | Methanoregula | 2 |
|  |  |  |  |  |  |  |  | Methanosphaerula | 1 |
|  |  |  |  |  |  |  |  | Methanospirillum | 1 |
|  |  |  |  |  |  |  |  | Methanosaeta | 3 |
|  |  |  |  |  |  |  |  | Methanococcoides | 1 |
|  |  |  |  |  |  |  |  | Methanohalobium | 1 |
|  |  |  |  |  |  |  |  | Methanohalophilus | 1 |
|  |  |  |  |  |  |  |  | Methanolobus | 2 |
|  |  |  |  |  |  |  |  | Methanomethylovorans | 1 |
|  |  |  |  |  |  |  |  | Methanosalsum | 1 |
|  |  |  |  |  |  |  |  | Methanosarcina | 4 |
|  |  |  |  |  |  |  |  | Methanopyrus | 1 |
|  |  |  |  |  |  |  |  | Pyrococcus | 6 |
|  |  |  |  |  |  |  |  | Thermococcus | 12 |

Table S3 Taxonomic classification of 2524 SecD sequences from bacteria used in this study

| Phylum |  | Class |  | Order |  | Family |  | Genus |  |
| --- | --- | --- | --- | --- | --- | --- | --- | --- | --- |
| Acidobacteria | 1 | Solibacteres | 1 | Solibacterales | 1 | Solibacteraceae | 1 | Candidatus Solibacter | 1 |
| Bacteroidetes | 206 | Bacteroidia | 147 | Bacteroidales | 147 | Bacteroidaceae | 68 | Bacteroides | 68 |
| Caldiserica | 1 | Cytophagia | 12 | Cytophagales | 12 | Porphyromonadaceae | 34 | Barnesiella | 2 |
| Chlorobi | 9 | Flavobacteriia | 38 | Flavobacteriales | 38 | Prevotellaceae | 43 | Coprobacter | 1 |
| Chloroflexi | 7 | Sphingobacteriia | 9 | Sphingobacteriales | 9 | Rikenellaceae | 2 | Odoribacter | 5 |
| Deinococcus-Thermus | 10 | Caldisericia | 1 | Caldisericales | 1 | Cyclobacteriaceae | 3 | Parabacteroides | 7 |
| Firmicutes | 469 | Chlorobia | 9 | Chlorobiales | 9 | Cytophagaceae | 8 | Porphyromonas | 11 |
| Ignavibacteriae | 1 | Anaerolineae | 1 | Anaerolineales | 1 | Flammeovirgaceae | 1 | Tannerella | 8 |
| Lentisphaerae | 1 | Caldilineae | 1 | Caldilineales | 1 | Blattabacteriaceae | 1 | Alloprevotella | 2 |
| Nitrospinae | 1 | Dehalococcoidia | 4 | Dehalococcoidales | 4 | Cryomorphaceae | 1 | Paraprevotella | 3 |
| Planctomycetes | 7 | Ktedonobacteria | 1 | Ktedonobacterales | 1 | Flavobacteriaceae | 36 | Prevotella | 38 |
| Proteobacteria | 1798 | Deinococci | 10 | Deinococcales | 4 | Saprospiraceae | 1 | Alistipes | 2 |
| Synergistetes | 7 | Bacilli | 170 | Thermales | 6 | Sphingobacteriaceae | 8 | Cyclobacterium | 2 |
| Verrucomicrobia | 6 | Clostridia | 241 | Bacillales | 170 | Caldisericaceae | 1 | Mariniradius | 1 |
|  |  | Erysipelotrichia | 3 | Clostridiales | 212 | Chlorobiaceae | 9 | Dyadobacter | 1 |
|  |  | Negativicutes | 55 | Halanaerobiales | 4 | Anaerolineaceae | 1 | Emticicia | 1 |
|  |  | Ignavibacteria | 1 | Natranaerobiales | 1 | Caldilineaceae | 1 | Fibrella | 1 |
|  |  | Lentisphaeria | 1 | Thermoanaerobacterales | 24 | Dehalococcoidaceae | 4 | Fibrisoma | 1 |
|  |  | Nitrospinia | 1 | Erysipelotrichales | 3 | Ktedonobacteraceae | 1 | Leadbetterella | 1 |
|  |  | Planctomycetia | 7 | Selenomonadales | 55 | Deinococcaceae | 3 | Rhodonellum | 1 |
|  |  | Alphaproteobacteria | 332 | Ignavibacteriales | 1 | Trueperaceae | 1 | Runella | 1 |
|  |  | Betaproteobacteria | 205 | Lentisphaerales | 1 | Thermaceae | 6 | Spirosoma | 1 |
|  |  | Deltaproteobacteria | 62 | Nitrospinales | 1 | Bacillaceae | 91 | Fulvivirga | 1 |
|  |  | Epsilonproteobacteria | 239 | Planctomycetales | 7 | Listeriaceae | 7 | Blattabacterium | 1 |
|  |  | Gammaproteobacteria | 959 | Caulobacterales | 14 | Paenibacillaceae | 24 | Fluviicola | 1 |
|  |  | Zetaproteobacteria | 1 | Magnetococcales | 1 | Planococcaceae | 3 | Bergeyella | 2 |
|  |  | Synergistia | 7 | Rhizobiales | 152 | Staphylococcaceae | 44 | Bizionia | 1 |
|  |  | Opitutae | 2 | Rhodobacterales | 56 | Thermoactinomycetaceae | 1 | Capnocytophaga | 12 |
|  |  | Verrucomicrobiae | 4 | Rhodospirillales | 36 | Clostridiaceae | 116 | Cellulophaga | 1 |
|  |  |  |  | Rickettsiales | 45 | Clostridiales Family XVII Incertae Sedis | 3 | Chryseobacterium | 2 |
|  |  |  |  | Sphingomonadales | 28 | Clostridiales Family XVIII Incertae Sedis | 1 | Elizabethkingia | 1 |
|  |  |  |  | Burkholderiales | 129 | Eubacteriaceae | 16 | Flavobacterium | 2 |
|  |  |  |  | Gallionellales | 2 | Heliobacteriaceae | 1 | Formosa | 1 |
|  |  |  |  | Hydrogenophilales | 2 | Lachnospiraceae | 35 | Krokinobacter | 1 |
|  |  |  |  | Methylophilales | 3 | Peptococcaceae | 18 | Lacinutrix | 1 |
|  |  |  |  | Neisseriales | 54 | Peptostreptococcaceae | 2 | Muricauda | 1 |
|  |  |  |  | Nitrosomonadales | 5 | Ruminococcaceae | 17 | Myroides | 6 |
|  |  |  |  | Rhodocyclales | 10 | Syntrophomonadaceae | 3 | Riemerella | 2 |
|  |  |  |  | Bdellovibrionales | 6 | Halanaerobiaceae | 2 | Weeksella | 1 |
|  |  |  |  | Desulfobacterales | 7 | Halobacteroidaceae | 2 | Winogradskyella | 1 |
|  |  |  |  | Desulfovibrionales | 20 | Natranaerobiaceae | 1 | Zobellia | 1 |
|  |  |  |  | Desulfurellales | 2 | Thermoanaerobacteraceae | 11 | Haliscomenobacter | 1 |
|  |  |  |  | Desulfuromonadales | 8 | Thermoanaerobacterales Family III Incertae Sedis | 10 | Arcticibacter | 1 |
|  |  |  |  | Myxococcales | 14 | Thermoanaerobacterales Family IV Incertae Sedis | 1 | Mucilaginibacter | 1 |
|  |  |  |  | Syntrophobacterales | 5 | Thermodesulfobiaceae | 2 | Pedobacter | 2 |
|  |  |  |  | Campylobacterales | 237 | Erysipelotrichaceae | 3 | Sphingobacterium | 4 |
|  |  |  |  | Nautiliales | 2 | Acidaminococcaceae | 10 | Caldisericum | 1 |
|  |  |  |  | Acidithiobacillales | 3 | Veillonellaceae | 45 | Chlorobium | 5 |
|  |  |  |  | Aeromonadales | 21 | Melioribacteraceae | 1 | Chloroherpeton | 1 |
|  |  |  |  | Alteromonadales | 107 | Lentisphaeraceae | 1 | Pelodictyon | 2 |
|  |  |  |  | Cardiobacteriales | 3 | Nitrospinaceae | 1 | Prosthecochloris | 1 |
|  |  |  |  | Chromatiales | 19 | Planctomycetaceae | 7 | Anaerolinea | 1 |
|  |  |  |  | Enterobacteriales | 259 | Caulobacteraceae | 14 | Caldilinea | 1 |
|  |  |  |  | Legionellales | 11 | Magnetococcaceae | 1 | Dehalococcoides | 4 |
|  |  |  |  | Methylococcales | 4 | Bartonellaceae | 24 | Ktedonobacter | 1 |
|  |  |  |  | Oceanospirillales | 20 | Beijerinckiaceae | 2 | Deinococcus | 3 |
|  |  |  |  | Pasteurellales | 69 | Bradyrhizobiaceae | 21 | Truepera | 1 |
|  |  |  |  | Pseudomonadales | 210 | Brucellaceae | 47 | Marinithermus | 1 |
|  |  |  |  | Salinisphaerales | 1 | Hyphomicrobiaceae | 4 | Meiothermus | 1 |
|  |  |  |  | Thiotrichales | 31 | Methylobacteriaceae | 10 | Thermus | 4 |
|  |  |  |  | Vibrionales | 160 | Methylocystaceae | 1 | Bacillus | 78 |
|  |  |  |  | Xanthomonadales | 41 | Phyllobacteriaceae | 8 | Caldalkalibacillus | 1 |
|  |  |  |  | Mariprofundales | 1 | Rhizobiaceae | 32 | Geobacillus | 9 |
|  |  |  |  | Synergistales | 7 | Xanthobacteraceae | 3 | Lysinibacillus | 2 |
|  |  |  |  | Puniceicoccales | 2 | Hyphomonadaceae | 4 | Virgibacillus | 1 |
|  |  |  |  | Verrucomicrobiales | 4 | Rhodobacteraceae | 52 | Listeria | 7 |
|  |  |  |  |  |  | Acetobacteraceae | 18 | Brevibacillus | 4 |
|  |  |  |  |  |  | Rhodospirillaceae | 18 | Paenibacillus | 20 |
|  |  |  |  |  |  | Anaplasmataceae | 19 | Paenisporosarcina | 1 |
|  |  |  |  |  |  | Candidatus Midichloriaceae | 1 | Planomicrobium | 1 |
|  |  |  |  |  |  | Rickettsiaceae | 25 | Solibacillus | 1 |
|  |  |  |  |  |  | Erythrobacteraceae | 2 | Staphylococcus | 44 |
|  |  |  |  |  |  | Sphingomonadaceae | 26 | Desmospora | 1 |
|  |  |  |  |  |  | Alcaligenaceae | 17 | Alkaliphilus | 1 |
|  |  |  |  |  |  | Burkholderiaceae | 70 | Butyricicoccus | 1 |
|  |  |  |  |  |  | Comamonadaceae | 21 | Caloramator | 1 |
|  |  |  |  |  |  | Oxalobacteraceae | 12 | Candidatus Arthromitus | 3 |
|  |  |  |  |  |  | Sutterellaceae | 9 | Clostridium | 110 |
|  |  |  |  |  |  | Gallionellaceae | 2 | Sulfobacillus | 1 |
|  |  |  |  |  |  | Hydrogenophilaceae | 2 | Thermaerobacter | 2 |
|  |  |  |  |  |  | Methylophilaceae | 3 | Symbiobacterium | 1 |
|  |  |  |  |  |  | Neisseriaceae | 54 | Acetobacterium | 1 |
|  |  |  |  |  |  | Nitrosomonadaceae | 5 | Eubacterium | 14 |
|  |  |  |  |  |  | Rhodocyclaceae | 10 | Pseudoramibacter | 1 |
|  |  |  |  |  |  | Bacteriovoracaceae | 3 | Heliobacterium | 1 |
|  |  |  |  |  |  | Bdellovibrionaceae | 3 | Anaerostipes | 1 |
|  |  |  |  |  |  | Desulfobacteraceae | 5 | Blautia | 8 |
|  |  |  |  |  |  | Desulfobulbaceae | 2 | Butyrivibrio | 1 |
|  |  |  |  |  |  | Desulfohalobiaceae | 1 | Catonella | 1 |
|  |  |  |  |  |  | Desulfomicrobiaceae | 1 | Cellulosilyticum | 2 |
|  |  |  |  |  |  | Desulfovibrionaceae | 18 | Coprococcus | 6 |
|  |  |  |  |  |  | Desulfurellaceae | 2 | Dorea | 5 |
|  |  |  |  |  |  | Desulfuromonadaceae | 1 | Lachnoanaerobaculum | 1 |
|  |  |  |  |  |  | Geobacteraceae | 5 | Marvinbryantia | 1 |
|  |  |  |  |  |  | Pelobacteraceae | 2 | Oribacterium | 4 |
|  |  |  |  |  |  | Cystobacteraceae | 2 | Roseburia | 3 |
|  |  |  |  |  |  | Kofleriaceae | 1 | Shuttleworthia | 1 |
|  |  |  |  |  |  | Myxococcaceae | 8 | Stomatobaculum | 1 |
|  |  |  |  |  |  | Nannocystaceae | 1 | Candidatus Desulforudis | 1 |
|  |  |  |  |  |  | Polyangiaceae | 2 | Dehalobacter | 1 |
|  |  |  |  |  |  | Syntrophaceae | 4 | Desulfitobacterium | 4 |
|  |  |  |  |  |  | Syntrophobacteraceae | 1 | Desulfosporosinus | 4 |
|  |  |  |  |  |  | Campylobacteraceae | 71 | Desulfotomaculum | 4 |
|  |  |  |  |  |  | Helicobacteraceae | 166 | Pelotomaculum | 1 |
|  |  |  |  |  |  | Nautiliaceae | 2 | Syntrophobotulus | 2 |
|  |  |  |  |  |  | Acidithiobacillaceae | 3 | Thermincola | 1 |
|  |  |  |  |  |  | Aeromonadaceae | 19 | Filifactor | 1 |
|  |  |  |  |  |  | Succinivibrionaceae | 2 | Peptoclostridium | 1 |
|  |  |  |  |  |  | Alteromonadaceae | 35 | Anaerotruncus | 2 |
|  |  |  |  |  |  | Colwelliaceae | 1 | Faecalibacterium | 2 |
|  |  |  |  |  |  | Ferrimonadaceae | 1 | Ruminococcus | 12 |
|  |  |  |  |  |  | Idiomarinaceae | 1 | Subdoligranulum | 1 |
|  |  |  |  |  |  | Moritellaceae | 2 | Dethiobacter | 1 |
|  |  |  |  |  |  | Pseudoalteromonadaceae | 31 | Syntrophomonas | 1 |
|  |  |  |  |  |  | Psychromonadaceae | 2 | Syntrophothermus | 1 |
|  |  |  |  |  |  | Shewanellaceae | 34 | Halanaerobium | 2 |
|  |  |  |  |  |  | Cardiobacteriaceae | 3 | Acetohalobium | 1 |
|  |  |  |  |  |  | Chromatiaceae | 11 | Halobacteroides | 1 |
|  |  |  |  |  |  | Ectothiorhodospiraceae | 7 | Natranaerobius | 1 |
|  |  |  |  |  |  | Halothiobacillaceae | 1 | Ammonifex | 1 |
|  |  |  |  |  |  | Enterobacteriaceae | 259 | Caldanaerobacter | 2 |
|  |  |  |  |  |  | Coxiellaceae | 5 | Carboxydothermus | 1 |
|  |  |  |  |  |  | Legionellaceae | 6 | Moorella | 1 |
|  |  |  |  |  |  | Methylococcaceae | 4 | Tepidanaerobacter | 1 |
|  |  |  |  |  |  | Alcanivoracaceae | 8 | Thermoanaerobacter | 5 |
|  |  |  |  |  |  | Hahellaceae | 1 | Caldicellulosiruptor | 5 |
|  |  |  |  |  |  | Halomonadaceae | 11 | Thermoanaerobacterium | 4 |
|  |  |  |  |  |  | Pasteurellaceae | 69 | Thermosediminibacter | 1 |
|  |  |  |  |  |  | Moraxellaceae | 92 | Mahella | 1 |
|  |  |  |  |  |  | Pseudomonadaceae | 118 | Coprothermobacter | 1 |
|  |  |  |  |  |  | Salinisphaeraceae | 1 | Thermodesulfobium | 1 |
|  |  |  |  |  |  | Francisellaceae | 17 | Bulleidia | 1 |
|  |  |  |  |  |  | Piscirickettsiaceae | 12 | Holdemania | 1 |
|  |  |  |  |  |  | Thiotrichaceae | 2 | Solobacterium | 1 |
|  |  |  |  |  |  | Vibrionaceae | 160 | Acidaminococcus | 7 |
|  |  |  |  |  |  | Sinobacteraceae | 1 | Phascolarctobacterium | 3 |
|  |  |  |  |  |  | Xanthomonadaceae | 40 | Acetonema | 1 |
|  |  |  |  |  |  | Mariprofundaceae | 1 | Anaeroglobus | 1 |
|  |  |  |  |  |  | Synergistaceae | 7 | Centipeda | 1 |
|  |  |  |  |  |  | Puniceicoccaceae | 2 | Dialister | 8 |
|  |  |  |  |  |  | Verrucomicrobia subdivision 3 | 1 | Megamonas | 1 |
|  |  |  |  |  |  | Verrucomicrobiaceae | 3 | Megasphaera | 6 |
|  |  |  |  |  |  |  |  | Mitsuokella | 1 |
|  |  |  |  |  |  |  |  | Pelosinus | 2 |
|  |  |  |  |  |  |  |  | Selenomonas | 12 |
|  |  |  |  |  |  |  |  | Sporomusa | 1 |
|  |  |  |  |  |  |  |  | Thermosinus | 1 |
|  |  |  |  |  |  |  |  | Veillonella | 10 |
|  |  |  |  |  |  |  |  | Melioribacter | 1 |
|  |  |  |  |  |  |  |  | Lentisphaera | 1 |
|  |  |  |  |  |  |  |  | Nitrospina | 1 |
|  |  |  |  |  |  |  |  | Isosphaera | 1 |
|  |  |  |  |  |  |  |  | Pirellula | 1 |
|  |  |  |  |  |  |  |  | Planctomyces | 4 |
|  |  |  |  |  |  |  |  | Rhodopirellula | 1 |
|  |  |  |  |  |  |  |  | Asticcacaulis | 7 |
|  |  |  |  |  |  |  |  | Brevundimonas | 3 |
|  |  |  |  |  |  |  |  | Caulobacter | 4 |
|  |  |  |  |  |  |  |  | Magnetococcus | 1 |
|  |  |  |  |  |  |  |  | Bartonella | 24 |
|  |  |  |  |  |  |  |  | Beijerinckia | 1 |
|  |  |  |  |  |  |  |  | Methylocella | 1 |
|  |  |  |  |  |  |  |  | Afipia | 4 |
|  |  |  |  |  |  |  |  | Bradyrhizobium | 11 |
|  |  |  |  |  |  |  |  | Nitrobacter | 1 |
|  |  |  |  |  |  |  |  | Oligotropha | 1 |
|  |  |  |  |  |  |  |  | Rhodopseudomonas | 4 |
|  |  |  |  |  |  |  |  | Brucella | 41 |
|  |  |  |  |  |  |  |  | Ochrobactrum | 6 |
|  |  |  |  |  |  |  |  | Hyphomicrobium | 3 |
|  |  |  |  |  |  |  |  | Pelagibacterium | 1 |
|  |  |  |  |  |  |  |  | Methylobacterium | 9 |
|  |  |  |  |  |  |  |  | Microvirga | 1 |
|  |  |  |  |  |  |  |  | Methylocystis | 1 |
|  |  |  |  |  |  |  |  | Chelativorans | 1 |
|  |  |  |  |  |  |  |  | Mesorhizobium | 7 |
|  |  |  |  |  |  |  |  | Agrobacterium | 6 |
|  |  |  |  |  |  |  |  | Candidatus Liberibacter | 2 |
|  |  |  |  |  |  |  |  | Ensifer | 1 |
|  |  |  |  |  |  |  |  | Rhizobium | 14 |
|  |  |  |  |  |  |  |  | Sinorhizobium | 9 |
|  |  |  |  |  |  |  |  | Azorhizobium | 1 |
|  |  |  |  |  |  |  |  | Starkeya | 1 |
|  |  |  |  |  |  |  |  | Xanthobacter | 1 |
|  |  |  |  |  |  |  |  | Hirschia | 1 |
|  |  |  |  |  |  |  |  | Hyphomonas | 2 |
|  |  |  |  |  |  |  |  | Oceanicaulis | 1 |
|  |  |  |  |  |  |  |  | Ahrensia | 1 |
|  |  |  |  |  |  |  |  | Celeribacter | 1 |
|  |  |  |  |  |  |  |  | Citreicella | 1 |
|  |  |  |  |  |  |  |  | Dinoroseobacter | 1 |
|  |  |  |  |  |  |  |  | Jannaschia | 1 |
|  |  |  |  |  |  |  |  | Ketogulonicigenium | 1 |
|  |  |  |  |  |  |  |  | Labrenzia | 3 |
|  |  |  |  |  |  |  |  | Leisingera | 1 |
|  |  |  |  |  |  |  |  | Loktanella | 2 |
|  |  |  |  |  |  |  |  | Maritimibacter | 1 |
|  |  |  |  |  |  |  |  | Oceanibulbus | 1 |
|  |  |  |  |  |  |  |  | Oceanicola | 2 |
|  |  |  |  |  |  |  |  | Oceaniovalibus | 1 |
|  |  |  |  |  |  |  |  | Paracoccus | 2 |
|  |  |  |  |  |  |  |  | Pelagibaca | 1 |
|  |  |  |  |  |  |  |  | Pseudovibrio | 1 |
|  |  |  |  |  |  |  |  | Rhodobacter | 7 |
|  |  |  |  |  |  |  |  | Rhodovulum | 1 |
|  |  |  |  |  |  |  |  | Roseibacterium | 1 |
|  |  |  |  |  |  |  |  | Roseivivax | 1 |
|  |  |  |  |  |  |  |  | Roseobacter | 7 |
|  |  |  |  |  |  |  |  | Roseovarius | 4 |
|  |  |  |  |  |  |  |  | Rubellimicrobium | 1 |
|  |  |  |  |  |  |  |  | Ruegeria | 5 |
|  |  |  |  |  |  |  |  | Sagittula | 1 |
|  |  |  |  |  |  |  |  | Salipiger | 1 |
|  |  |  |  |  |  |  |  | Sulfitobacter | 1 |
|  |  |  |  |  |  |  |  | Thalassobium | 1 |
|  |  |  |  |  |  |  |  | Acetobacter | 5 |
|  |  |  |  |  |  |  |  | Acidiphilium | 1 |
|  |  |  |  |  |  |  |  | Asaia | 1 |
|  |  |  |  |  |  |  |  | Gluconacetobacter | 3 |
|  |  |  |  |  |  |  |  | Gluconobacter | 4 |
|  |  |  |  |  |  |  |  | Granulibacter | 2 |
|  |  |  |  |  |  |  |  | Komagataeibacter | 1 |
|  |  |  |  |  |  |  |  | Roseomonas | 1 |
|  |  |  |  |  |  |  |  | Azospirillum | 5 |
|  |  |  |  |  |  |  |  | Candidatus Endolissoclinum | 2 |
|  |  |  |  |  |  |  |  | Magnetospirillum | 1 |
|  |  |  |  |  |  |  |  | Oceanibaculum | 1 |
|  |  |  |  |  |  |  |  | Phaeospirillum | 2 |
|  |  |  |  |  |  |  |  | Rhodospirillum | 4 |
|  |  |  |  |  |  |  |  | Skermanella | 1 |
|  |  |  |  |  |  |  |  | Thalassospira | 1 |
|  |  |  |  |  |  |  |  | Tistrella | 1 |
|  |  |  |  |  |  |  |  | Anaplasma | 5 |
|  |  |  |  |  |  |  |  | Ehrlichia | 4 |
|  |  |  |  |  |  |  |  | Neorickettsia | 2 |
|  |  |  |  |  |  |  |  | Wolbachia | 8 |
|  |  |  |  |  |  |  |  | Candidatus Midichloria | 1 |
|  |  |  |  |  |  |  |  | Orientia | 1 |
|  |  |  |  |  |  |  |  | Rickettsia | 24 |
|  |  |  |  |  |  |  |  | Erythrobacter | 2 |
|  |  |  |  |  |  |  |  | Blastomonas | 1 |
|  |  |  |  |  |  |  |  | Novosphingobium | 6 |
|  |  |  |  |  |  |  |  | Sphingobium | 9 |
|  |  |  |  |  |  |  |  | Sphingomonas | 5 |
|  |  |  |  |  |  |  |  | Sphingopyxis | 1 |
|  |  |  |  |  |  |  |  | Zymomonas | 4 |
|  |  |  |  |  |  |  |  | Achromobacter | 5 |
|  |  |  |  |  |  |  |  | Alcaligenes | 1 |
|  |  |  |  |  |  |  |  | Bordetella | 5 |
|  |  |  |  |  |  |  |  | Castellaniella | 1 |
|  |  |  |  |  |  |  |  | Pelistega | 1 |
|  |  |  |  |  |  |  |  | Pusillimonas | 1 |
|  |  |  |  |  |  |  |  | Taylorella | 3 |
|  |  |  |  |  |  |  |  | Burkholderia | 44 |
|  |  |  |  |  |  |  |  | Candidatus Glomeribacter | 1 |
|  |  |  |  |  |  |  |  | Cupriavidus | 6 |
|  |  |  |  |  |  |  |  | Lautropia | 1 |
|  |  |  |  |  |  |  |  | Limnobacter | 1 |
|  |  |  |  |  |  |  |  | Pandoraea | 3 |
|  |  |  |  |  |  |  |  | Polynucleobacter | 2 |
|  |  |  |  |  |  |  |  | Ralstonia | 12 |
|  |  |  |  |  |  |  |  | Acidovorax | 5 |
|  |  |  |  |  |  |  |  | Alicycliphilus | 1 |
|  |  |  |  |  |  |  |  | Comamonas | 1 |
|  |  |  |  |  |  |  |  | Curvibacter | 1 |
|  |  |  |  |  |  |  |  | Delftia | 4 |
|  |  |  |  |  |  |  |  | Hydrogenophaga | 1 |
|  |  |  |  |  |  |  |  | Hylemonella | 1 |
|  |  |  |  |  |  |  |  | Polaromonas | 3 |
|  |  |  |  |  |  |  |  | Ramlibacter | 1 |
|  |  |  |  |  |  |  |  | Variovorax | 2 |
|  |  |  |  |  |  |  |  | Verminephrobacter | 1 |
|  |  |  |  |  |  |  |  | Collimonas | 1 |
|  |  |  |  |  |  |  |  | Herbaspirillum | 4 |
|  |  |  |  |  |  |  |  | Herminiimonas | 1 |
|  |  |  |  |  |  |  |  | Janthinobacterium | 4 |
|  |  |  |  |  |  |  |  | Oxalobacter | 2 |
|  |  |  |  |  |  |  |  | Parasutterella | 2 |
|  |  |  |  |  |  |  |  | Sutterella | 7 |
|  |  |  |  |  |  |  |  | Gallionella | 1 |
|  |  |  |  |  |  |  |  | Sideroxydans | 1 |
|  |  |  |  |  |  |  |  | Sulfuricella | 1 |
|  |  |  |  |  |  |  |  | Thiobacillus | 1 |
|  |  |  |  |  |  |  |  | Methylobacillus | 1 |
|  |  |  |  |  |  |  |  | Methylotenera | 1 |
|  |  |  |  |  |  |  |  | Methylovorus | 1 |
|  |  |  |  |  |  |  |  | Chromobacterium | 1 |
|  |  |  |  |  |  |  |  | Eikenella | 2 |
|  |  |  |  |  |  |  |  | Kingella | 3 |
|  |  |  |  |  |  |  |  | Laribacter | 1 |
|  |  |  |  |  |  |  |  | Neisseria | 44 |
|  |  |  |  |  |  |  |  | Pseudogulbenkiania | 2 |
|  |  |  |  |  |  |  |  | Simonsiella | 1 |
|  |  |  |  |  |  |  |  | Nitrosomonas | 3 |
|  |  |  |  |  |  |  |  | Nitrosospira | 2 |
|  |  |  |  |  |  |  |  | Aromatoleum | 1 |
|  |  |  |  |  |  |  |  | Azoarcus | 1 |
|  |  |  |  |  |  |  |  | Azospira | 1 |
|  |  |  |  |  |  |  |  | Dechloromonas | 1 |
|  |  |  |  |  |  |  |  | Methyloversatilis | 1 |
|  |  |  |  |  |  |  |  | Sulfuritalea | 1 |
|  |  |  |  |  |  |  |  | Thauera | 4 |
|  |  |  |  |  |  |  |  | Bacteriovorax | 3 |
|  |  |  |  |  |  |  |  | Bdellovibrio | 3 |
|  |  |  |  |  |  |  |  | Desulfobacter | 1 |
|  |  |  |  |  |  |  |  | Desulfobacula | 1 |
|  |  |  |  |  |  |  |  | Desulfococcus | 2 |
|  |  |  |  |  |  |  |  | Desulfotignum | 1 |
|  |  |  |  |  |  |  |  | Desulfotalea | 1 |
|  |  |  |  |  |  |  |  | Desulfurivibrio | 1 |
|  |  |  |  |  |  |  |  | Desulfohalobium | 1 |
|  |  |  |  |  |  |  |  | Desulfomicrobium | 1 |
|  |  |  |  |  |  |  |  | Bilophila | 3 |
|  |  |  |  |  |  |  |  | Desulfovibrio | 14 |
|  |  |  |  |  |  |  |  | Lawsonia | 1 |
|  |  |  |  |  |  |  |  | Desulfurella | 1 |
|  |  |  |  |  |  |  |  | Hippea | 1 |
|  |  |  |  |  |  |  |  | Desulfuromonas | 1 |
|  |  |  |  |  |  |  |  | Geobacter | 5 |
|  |  |  |  |  |  |  |  | Pelobacter | 2 |
|  |  |  |  |  |  |  |  | Cystobacter | 1 |
|  |  |  |  |  |  |  |  | Stigmatella | 1 |
|  |  |  |  |  |  |  |  | Haliangium | 1 |
|  |  |  |  |  |  |  |  | Anaeromyxobacter | 4 |
|  |  |  |  |  |  |  |  | Corallococcus | 1 |
|  |  |  |  |  |  |  |  | Myxococcus | 3 |
|  |  |  |  |  |  |  |  | Plesiocystis | 1 |
|  |  |  |  |  |  |  |  | Sorangium | 2 |
|  |  |  |  |  |  |  |  | Desulfobacca | 1 |
|  |  |  |  |  |  |  |  | Desulfomonile | 2 |
|  |  |  |  |  |  |  |  | Syntrophus | 1 |
|  |  |  |  |  |  |  |  | Syntrophobacter | 1 |
|  |  |  |  |  |  |  |  | Arcobacter | 6 |
|  |  |  |  |  |  |  |  | Campylobacter | 62 |
|  |  |  |  |  |  |  |  | Sulfurospirillum | 3 |
|  |  |  |  |  |  |  |  | Helicobacter | 158 |
|  |  |  |  |  |  |  |  | Sulfuricurvum | 2 |
|  |  |  |  |  |  |  |  | Sulfurimonas | 4 |
|  |  |  |  |  |  |  |  | Thiovulum | 1 |
|  |  |  |  |  |  |  |  | Wolinella | 1 |
|  |  |  |  |  |  |  |  | Caminibacter | 1 |
|  |  |  |  |  |  |  |  | Nautilia | 1 |
|  |  |  |  |  |  |  |  | Acidithiobacillus | 3 |
|  |  |  |  |  |  |  |  | Aeromonas | 18 |
|  |  |  |  |  |  |  |  | Tolumonas | 1 |
|  |  |  |  |  |  |  |  | Succinatimonas | 2 |
|  |  |  |  |  |  |  |  | Agarivorans | 2 |
|  |  |  |  |  |  |  |  | Alishewanella | 2 |
|  |  |  |  |  |  |  |  | Alteromonas | 7 |
|  |  |  |  |  |  |  |  | Glaciecola | 11 |
|  |  |  |  |  |  |  |  | Marinobacter | 12 |
|  |  |  |  |  |  |  |  | Saccharophagus | 1 |
|  |  |  |  |  |  |  |  | Colwellia | 1 |
|  |  |  |  |  |  |  |  | Ferrimonas | 1 |
|  |  |  |  |  |  |  |  | Idiomarina | 1 |
|  |  |  |  |  |  |  |  | Moritella | 2 |
|  |  |  |  |  |  |  |  | Pseudoalteromonas | 31 |
|  |  |  |  |  |  |  |  | Psychromonas | 2 |
|  |  |  |  |  |  |  |  | Shewanella | 34 |
|  |  |  |  |  |  |  |  | Cardiobacterium | 2 |
|  |  |  |  |  |  |  |  | Dichelobacter | 1 |
|  |  |  |  |  |  |  |  | Marichromatium | 1 |
|  |  |  |  |  |  |  |  | Nitrosococcus | 2 |
|  |  |  |  |  |  |  |  | Rheinheimera | 3 |
|  |  |  |  |  |  |  |  | Thiocapsa | 1 |
|  |  |  |  |  |  |  |  | Thioflavicoccus | 1 |
|  |  |  |  |  |  |  |  | Thiorhodococcus | 2 |
|  |  |  |  |  |  |  |  | Thiorhodovibrio | 1 |
|  |  |  |  |  |  |  |  | Alkalilimnicola | 1 |
|  |  |  |  |  |  |  |  | Halorhodospira | 2 |
|  |  |  |  |  |  |  |  | Spiribacter | 1 |
|  |  |  |  |  |  |  |  | Thioalkalivibrio | 2 |
|  |  |  |  |  |  |  |  | Thiorhodospira | 1 |
|  |  |  |  |  |  |  |  | Halothiobacillus | 1 |
|  |  |  |  |  |  |  |  | Arsenophonus | 1 |
|  |  |  |  |  |  |  |  | Brenneria | 1 |
|  |  |  |  |  |  |  |  | Candidatus Blochmannia | 2 |
|  |  |  |  |  |  |  |  | Candidatus Ishikawaella | 1 |
|  |  |  |  |  |  |  |  | Candidatus Hamiltonella | 1 |
|  |  |  |  |  |  |  |  | Candidatus Regiella | 1 |
|  |  |  |  |  |  |  |  | Cedecea | 1 |
|  |  |  |  |  |  |  |  | Citrobacter | 5 |
|  |  |  |  |  |  |  |  | Cronobacter | 10 |
|  |  |  |  |  |  |  |  | Dickeya | 2 |
|  |  |  |  |  |  |  |  | Edwardsiella | 4 |
|  |  |  |  |  |  |  |  | Enterobacter | 24 |
|  |  |  |  |  |  |  |  | Erwinia | 8 |
|  |  |  |  |  |  |  |  | Escherichia | 62 |
|  |  |  |  |  |  |  |  | Hafnia | 1 |
|  |  |  |  |  |  |  |  | Klebsiella | 17 |
|  |  |  |  |  |  |  |  | Kosakonia | 1 |
|  |  |  |  |  |  |  |  | Morganella | 2 |
|  |  |  |  |  |  |  |  | Pantoea | 11 |
|  |  |  |  |  |  |  |  | Pectobacterium | 4 |
|  |  |  |  |  |  |  |  | Photorhabdus | 2 |
|  |  |  |  |  |  |  |  | Plesiomonas | 1 |
|  |  |  |  |  |  |  |  | Proteus | 3 |
|  |  |  |  |  |  |  |  | Providencia | 8 |
|  |  |  |  |  |  |  |  | Rahnella | 2 |
|  |  |  |  |  |  |  |  | Raoultella | 1 |
|  |  |  |  |  |  |  |  | Salmonella | 36 |
|  |  |  |  |  |  |  |  | Serratia | 15 |
|  |  |  |  |  |  |  |  | Shigella | 5 |
|  |  |  |  |  |  |  |  | Sodalis | 2 |
|  |  |  |  |  |  |  |  | Xenorhabdus | 3 |
|  |  |  |  |  |  |  |  | Yersinia | 22 |
|  |  |  |  |  |  |  |  | Coxiella | 4 |
|  |  |  |  |  |  |  |  | Rickettsiella | 1 |
|  |  |  |  |  |  |  |  | Legionella | 6 |
|  |  |  |  |  |  |  |  | Methylobacter | 1 |
|  |  |  |  |  |  |  |  | Methylococcus | 1 |
|  |  |  |  |  |  |  |  | Methyloglobulus | 1 |
|  |  |  |  |  |  |  |  | Methylomicrobium | 1 |
|  |  |  |  |  |  |  |  | Alcanivorax | 7 |
|  |  |  |  |  |  |  |  | Kangiella | 1 |
|  |  |  |  |  |  |  |  | Hahella | 1 |
|  |  |  |  |  |  |  |  | Halomonas | 11 |
|  |  |  |  |  |  |  |  | Actinobacillus | 13 |
|  |  |  |  |  |  |  |  | Aggregatibacter | 11 |
|  |  |  |  |  |  |  |  | Bibersteinia | 3 |
|  |  |  |  |  |  |  |  | Gallibacterium | 1 |
|  |  |  |  |  |  |  |  | Haemophilus | 28 |
|  |  |  |  |  |  |  |  | Histophilus | 2 |
|  |  |  |  |  |  |  |  | Mannheimia | 7 |
|  |  |  |  |  |  |  |  | Pasteurella | 4 |
|  |  |  |  |  |  |  |  | Acinetobacter | 79 |
|  |  |  |  |  |  |  |  | Enhydrobacter | 1 |
|  |  |  |  |  |  |  |  | Moraxella | 2 |
|  |  |  |  |  |  |  |  | Psychrobacter | 10 |
|  |  |  |  |  |  |  |  | Azotobacter | 1 |
|  |  |  |  |  |  |  |  | Cellvibrio | 2 |
|  |  |  |  |  |  |  |  | Pseudomonas | 115 |
|  |  |  |  |  |  |  |  | Salinisphaera | 1 |
|  |  |  |  |  |  |  |  | Francisella | 17 |
|  |  |  |  |  |  |  |  | Cycloclasticus | 2 |
|  |  |  |  |  |  |  |  | Methylophaga | 6 |
|  |  |  |  |  |  |  |  | Piscirickettsia | 1 |
|  |  |  |  |  |  |  |  | Thioalkalimicrobium | 2 |
|  |  |  |  |  |  |  |  | Thiomicrospira | 1 |
|  |  |  |  |  |  |  |  | Beggiatoa | 2 |
|  |  |  |  |  |  |  |  | Aliivibrio | 6 |
|  |  |  |  |  |  |  |  | Grimontia | 3 |
|  |  |  |  |  |  |  |  | Photobacterium | 15 |
|  |  |  |  |  |  |  |  | Vibrio | 136 |
|  |  |  |  |  |  |  |  | Hydrocarboniphaga | 1 |
|  |  |  |  |  |  |  |  | Frateuria | 1 |
|  |  |  |  |  |  |  |  | Pseudoxanthomonas | 1 |
|  |  |  |  |  |  |  |  | Rhodanobacter | 5 |
|  |  |  |  |  |  |  |  | Stenotrophomonas | 8 |
|  |  |  |  |  |  |  |  | Wohlfahrtiimonas | 1 |
|  |  |  |  |  |  |  |  | Xanthomonas | 20 |
|  |  |  |  |  |  |  |  | Xylella | 4 |
|  |  |  |  |  |  |  |  | Mariprofundus | 1 |
|  |  |  |  |  |  |  |  | Aminobacterium | 1 |
|  |  |  |  |  |  |  |  | Anaerobaculum | 1 |
|  |  |  |  |  |  |  |  | Dethiosulfovibrio | 1 |
|  |  |  |  |  |  |  |  | Jonquetella | 1 |
|  |  |  |  |  |  |  |  | Pyramidobacter | 1 |
|  |  |  |  |  |  |  |  | Thermanaerovibrio | 1 |
|  |  |  |  |  |  |  |  | Thermovirga | 1 |
|  |  |  |  |  |  |  |  | Coraliomargarita | 2 |
|  |  |  |  |  |  |  |  | Pedosphaera | 1 |
|  |  |  |  |  |  |  |  | Akkermansia | 3 |
